# Efficacy and safety of PD-1 inhibitors plus chemotherapy for advanced unresectable gastroesophageal cancer: a systematic review and meta-analysis of phase III randomized controlled trials

**DOI:** 10.3389/fonc.2026.1838929

**Published:** 2026-09-22

**Authors:** Chenfeng Hua, Rui Zhang, Xiuli Mo, Quanping Yan, Chengjie Ma, Fuwei Xie, Yongliang Jia

**Affiliations:** 1 Key Laboratory of Tobacco Chemistry, Zhengzhou Tobacco Research Institute of CNTC, Zhengzhou, China; 2 Beijing Life Science Academy, Beijing, China; 3 Department of Oncology, the First Affiliated Hospital of Zhengzhou University, Zhengzhou, China; 4 Henan Institute of Medical and Pharmaceutical Sciences, Zhengzhou University, Zhengzhou, China; 5 Henan Provincial Engineering Technology Research Center for Smart Health Care, Zhengzhou University of Industrial Technology, Zhengzhou, China

**Keywords:** PD-1 inhibitors, first-line therapy, immunotherapeutic agents, meta-analysis, randomized controlled trials

## Abstract

**Objective:**

The FDA approval of immune checkpoint inhibitors for advanced gastroesophageal cancer (GEC) independent of programmed death-ligand 1 (PD-L1) expression status has sparked notable clinical controversy, highlighting the urgent need for evidence-based evaluation of the efficacy and safety of various PD-1 inhibitors combinations for this malignancy.

**Methods:**

We followed the pre-registered PROSPERO protocol (CRD42022325401) to search the databases and conferences. The primary outcomes included overall survival (OS), progression-free survival (PFS), and treatment-related adverse event (TRAE). A random-effects model was applied for meta-analysis, with statistical data analysis performed using Review Manager software and the distribution of adverse event (AE) subtypes visualized via R software.

**Results:**

A total of 6625 patients from 9 phase III randomized controlled trials (RCTs) were ultimately included in this analysis. The overall risk of included RCTs was low. Results found immunotherapy combined with chemotherapy improved patients’ prognosis more than single-agent chemotherapy, with corresponding increased but manageable adverse effects. Subgroup analyses by drug type further revealed that sintilimab in combination with chemotherapy exhibited more efficacy than other PD-1 inhibitor-based regimens. The survival benefit in the immunotherapy combined with chemotherapy group compared to chemotherapy alone was seen in both PD-L1 CPS positive and TPS positive. The evidence strength was moderate.

**Conclusion:**

Findings from this comprehensive study confirm that immunotherapy combined with chemotherapy is more efficacious than traditional chemotherapy in the first-line treatment of patients with advanced unresectable GEC. Sintilimab combined with chemotherapy demonstrated favorable efficacy and safety profiles compared with other PD-1 therapies in terms of OS and PFS benefit without increasing TRAE base on indirect comparisons. The moderate evidence indicated PD-1 combined with chemotherapy may be a viable option for advanced unresectable GEC.

**Systematic review registration:**

https://www.crd.york.ac.uk/PROSPERO/, identifier CRD42022325401.

## 1 Introduction

Advanced esophageal cancer (AEC), gastroesophageal junction carcinoma (GEJC), and gastric adenocarcinoma (GAC) are regarded as clinically interchangeable for systemic therapy selection, a position endorsed by the 2022 National Comprehensive Cancer Network (NCCN) guidelines (1). Gastric cancer ranks fifth among malignancy worldwide (2). The diagnosis of gastric cancer is often at an advanced stage and gastric cancer is the third leading cause of cancer death (2). Gastric cancer is anatomically divided into adenocarcinoma of the stomach, and adenocarcinoma of the gastroesophageal junction. Adenocarcinomas of the distal esophagus and gastroesophageal junction (GEJ) have historically been categorized alongside gastric cancer; this anatomical and biological continuum renders their systemic treatments particularly relevant to integrated therapeutic strategies (3). Although there are some advances in treatment over the last few decades, the 5-year survival rate of gastric cancer remains low (4, 5). The stage at diagnosis has a significant influence on patients’ prognosis for gastric cancer, and 35% of patients are diagnosed with advanced stage (6). For locally advanced or relapsed or metastatic disease in the HER2-negative population, fluorouracil combined with oxaliplatin and nivolumab is recommended as first-line therapy in the tumor of PD-L1 combined positive score (CPS) no less than 5 (1, 7).

Preclinical and clinical data suggest that chemotherapy may augment anti-tumor immunity (8–10). Potential mechanisms include immunostimulatory factors released from dying tumor cells, and off-target effects on distinct immune cell subsets (8–10). The FDA has approved nivolumab in combination with chemotherapy for AEC, GEJC, and AGC. This approval was based on the benefit of survival in several phase III clinical studies (7, 11, 12). The National Medical Products Administration (NMPA) of China approved sintilimab combined with fluorouracil- and platinum-based chemotherapy for unresectable locally advanced, recurrent, recurrent, or metastatic GC and GEJC. The approval was based on a randomized controlled phase III clinical trial ORIENT-16, which compared sintilimab combined with chemotherapy to chemotherapy (13). ORIENT-16 found that sintilimab combined with chemotherapy brought significant benefits to both the PD-L1 CPS ≥5 population and the general population. Another clinical trial the ORIENT-15 also indicated a better OS benefit of sintilimab in combination with chemotherapy compared with chemotherapy alone for patients with locally advanced or metastatic ESCC (14). Furthermore, according to the clinical studies KEYNOTE-590, E SCORT-1st, and JUPITER-06, chemotherapy combined with pembrolizumab, camrelizumab, and toripalimab is demonstrated clinical efficacy compared with chemotherapy alone in terms of survival (15–17).

A prior meta-analysis (18) of prospective clinical studies demonstrated that PD-1/PD-L1 inhibitors therapy confers effective activity and manageable adverse event (AE) in treating AGC and GEJC. PD-L1 overexpression correlated with higher objective response rates (ORR). A meta-analysis (19) of gastroesophageal cancer comparing immune checkpoint inhibitors (ICIs) plus conventional therapy with ICIs monotherapy showed that combined therapies improved tumor response rate, prolonged progression-free survival (PFS), and overall survival (OS). Another meta-analysis (20) compared ICIs plus chemotherapy to chemotherapy alone in AEC. Toripalimab plus chemotherapy conferred the greatest OS benefit, whereas sintilimab plus chemotherapy and camrelizumab plus chemotherapy yielded the best PFS. Patients who received nivolumab in combination with chemotherapy improved the most in terms of objective response rate (ORR).

However, none study has been conducted to compare various immune-chemotherapy combinations in the first-line treatment of gastroesophageal cancer by evaluating the evidence strength following the Grading of Recommendations Assessment, Development, and Evaluations (GRADE) approach. Thus, to identify the best combination strategies for adult patients with advanced gastroesophageal cancers, a systematic review and meta-analysis were carried out, in which different PD-1 inhibitors may be compared through subgroup analyses or indirect methods.

## 2 Method

This meta-analysis followed the pre-specified PROSPERO registered protocol (CRD42022325401). All procedures were carried out following the PRISMA 2020 checklist, including eligibility criteria, search strategy, risk of bias assessment, certainty assessment and results interpretation (**Supplementary Table 1**).

### 2.1 Data sources and search strategy

The databases including PubMed, EMBA SE, Cochrane Library, and ClinicalTrials.gov were systematically searched for relevant studies conducted between 1 January 2010, and 1 January 2024. To include the most recent results, online proceedings from annual conferences between 2020 and 2024 were also consulted. The European Society of Medical Oncology (ESMO), American Society of Clinical Oncology (ASCO), and the American Association for Cancer Research (AACR) were also searched. The search words were “CTLA-4 or PD-L1 or PD-1 or nivolumab or pembrolizumab or camrelizumab or sintilimab or tislelizumab or toripalimab or penpulimab or atezolizumab or durvalumab or avelumab or tremelimumab or ipilimumab”, “chemotherapy”, “stomach neoplasms or esophageal neoplasms or esophagogastric junction” and “trial or randomized controlled trial” (**Supplementary S1** shows more details of search strategy).

### 2.2 Selection criteria

Inclusion criteria including

1. Randomized phase 3 clinical trial;
2. All the patients had histologically or cytologically confirmed advanced gastroesophageal cancer;
3. Studies comparing the survival advantages and safety of PD-1 inhibitors combined with chemotherapy versus chemotherapy (plus placebo) for advanced gastroesophageal cancer;
4. Studies with the first-line treatment;

Exclusion criteria including

1. Clinical trials did not report sufficient data;
2. Single-arm studies, retrospective studies, case reports, not first-line treatment studies, and others;
3. PD-L1 inhibitors or CTLA-4 inhibitors. This study excluded PD-L1 inhibitors because they exhibit distinct mechanistic differences from PD-1 inhibitors (e.g., PD-1 inhibitors block interactions with both PD-L1 and PD-L2, whereas PD-L1 inhibitors only block PD-L1 but retain PD-L2 binding; additionally, they differ in Fc-domain engineering and ADCC/CDC effects). These differences could introduce substantial clinical and biological heterogeneity. CTLA-4 inhibitors were excluded because they target a completely different phase of the immune response (the priming phase in the lymph nodes rather than the effector phase in the tumor microenvironment) and have distinct toxicity profiles. Restricting the analysis to PD-1 inhibitors ensures a more homogeneous and biologically comparable intervention group.
4. Obsolete control regimens, such as chemotherapy monotherapy (e.g., 5-Fluorouracil or cisplatin alone) or outdated triplets (e.g., Epirubicin, Cisplatin, and Fluorouracil or Docetaxel, Cisplatin, and Fluorouracil);
5. Investigational agents that failed to meet primary endpoints in Phase 3 trials or were officially terminated/rejected by regulatory bodies;
6. Insufficient data or duplicate publications.

### 2.3 Data extraction and quality assessment

Two authors independently carried out data extraction and quality assessment. The following primary study information was extracted: study names, NCT numbers, experiment arms, control arms, and clinicopathological characteristics of patients. The primary efficacy outcome indicators were overall survival (OS), progression-free survival (PFS), objective response rate (ORR), treatment-related adverse event (TRAE), and serious adverse event (SAE). The mean deviations (MDs), hazard ratios (HRs) and 95% confidence intervals (CIs) for OS and PFS, in which pooled hazard ratios were the primary endpoint for time-to-event analyses; pooled absolute time differences were reported only as an exploratory descriptive measure of absolute benefit, as well as the odds ratio (OR) and 95% CI for ORR, were calculated. The safety data extracted included any grade of AE, AE above grade 3, medication interruption and death due to AE, and immune-related AE (IRAE). Since two scoring systems are commonly used for PD-L1 status, the TPS score is defined as the percentage of tumor cells with any intensity of PD-L1 membrane staining; the CPS score was defined as the sum of the number of PD-L1 stained tumor cells and tumor-associated immune cells per 100 tumor cells (21). Therefore, data on the different scoring methods to investigate whether there are differences in assessing efficacy was collected.

The Cochrane Risk of Bias (RoB) 2 Tool was used to assess the quality of included RCTs (22), which included the following five domains: bias resulting from the randomization process, bias resulting from interventions that differ from those intended, bias resulting from missing outcome data, bias resulting from the measurement of the outcome, and bias resulting from the choice of the reported result. The overall risk of bias for each included RCT was then calculated (23). The RoB of included RCTs was then divided into three levels: low risk, high risk, and some concerns.

### 2.4 Statistical analysis and bias assessment

The primary purpose of this study was to compare the outcomes (OS, PFS, ORR, TRAE, and SAE) of immunotherapy combination chemotherapy with chemotherapy alone or combination placebo. A pairwise meta-analysis based on a random-effects model with frequentist inference was conducted for statistical data synthesis. Given concerns regarding transitivity and network connectivity, this study did not carry out a network meta-analysis. Potential substantial heterogeneity across included trials, driven by varied control-arm chemotherapy backbones, different cut-off thresholds, and distinct patient populations, would render the core assumptions for a valid NMA invalid. OS and PFS were demonstrated as hazard ratio (HR) and mean differences (MD) with their 95% confidence intervals (CI) using the inverse-variance method. The ORR and AE frequency were calculated using odds ratio (ORs) with the 95% CIs were pooled using the Mantel-Haenszel method. The Cochrane Q test and the I^2^ statistic were used to assess RCT heterogeneity. The risk of publication bias was assessed using visual inspection of funnel plots. Subgroup analysis was used to explore the sources of data heterogeneity. The distributions of the various types of TRAR and IRAE are represented by bubble plots and box line plots, which were plotted with the R package “ggplot”. Review Manager 5.4.1 was used to conduct the statistical analyses. Every test was two-sided, and statistical significance was defined as a P-value less than 0.05.

### 2.5 GRADE evidence strength

The Grade of Recommendations, Assessment, Development, and Evaluation (GRADE) evidence system was used to assess the evidence level of the meta-analysis, which is divided into four categories: high, medium, low, and very low (24). GRADE assessment was performed independently by two investigators. Any disagreements between the two investigators were resolved through discussion to reach a consensus, or by consulting a third senior investigator when necessary.

## 3 Results

### 3.1 Characteristics of included clinical trials

Following a thorough revision, 9 RCTs with a total of 6625 patients were included (**Figure 1**). The results of ORIENT-16 were reported on 2021 ESMO and the other 8 studies were searched from PubMed. All studies were phase 3 clinical trials that compared immunotherapy plus chemotherapy to chemotherapy in patients with advanced gastroesophageal cancer who were previously untreated. **Table 1** summarizes the main characteristics of the patients. All nine studies used PD-1 inhibitors: pembrolizumab in two RCTs, camrelizumab in one RCT, nivolumab in three RCTs, sintilimab in two RCTs, and toripalimab in one RCT. Patients with PD-L1 CPS values larger than or equal to 5 were included in the CheckMate 648 study, and all patients recruited in KEYNOTE-062 had PD-L1 CPS values greater than or equal to 1.

**Figure 1:**
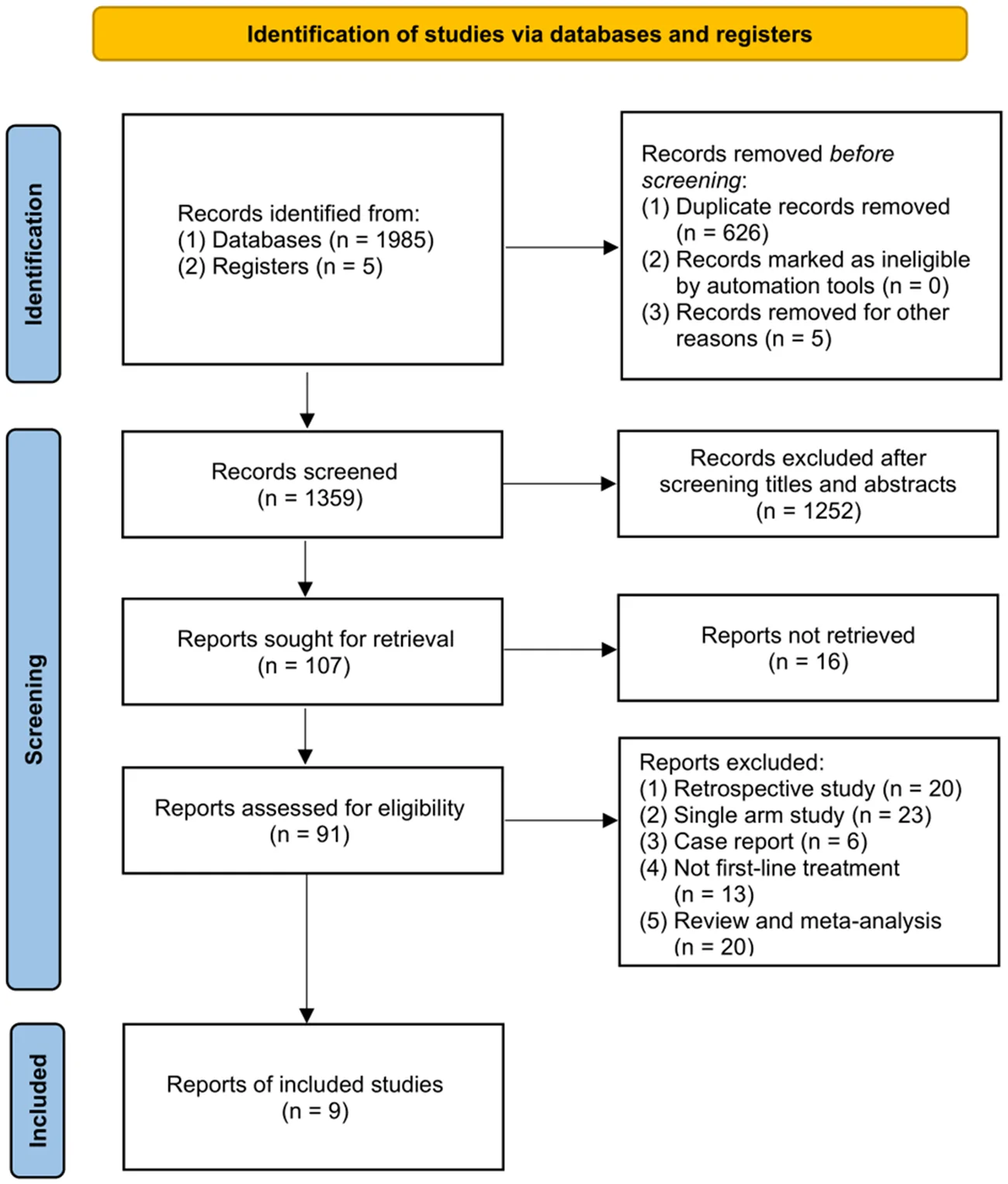
The PRISMA flowchart of the study selection process for the meta-analysis.

**Table 1:** Baseline characteristics of RTCs included in the meta-analysis.

Study phase, design	Source (publication year)	Registered ID (randomization)	Sample size intervention/control (median age)	(Male/female)	Histology	Ethnicity (%)	Intervention arm (s)	Control arm (s)
KEYNOTE-590 (III, double-blind)	Lancet (2021)	NCT03189719 (1:1)	373/376 (64/62)	(625/124)	E/GEJ	Asian (53.4) White (37.1)	Pembrolizumab + chemotherapy (5-fluorouracil 800 mg/m² on d1-5 + cisplatin 80 mg/m² on d1)	Placebo + chemotherapy (5-fluorouracil 800 mg/m² on d1-5 + cisplatin 80 mg/m² on d1)
KEYNOTE-062 (III, partially blinded)	JAMA Onco (2020)	NCT02494583 (1:1:1)*	257/250 (62/62.5)	(374/133)	G/GEJ	Asian (24.7) White (58.2)	Pembrolizumab + chemotherapy (cisplatin 80mg/m^2^)/d d1 + fluorouracil 800 mg/m^2^/d d1–5 or capecitabine 1000 mg/m^2^ bid d1-14)	Placebo + chemotherapy (cisplatin 80 mg/m^2^/d d1 + fluorouracil 800 mg/m^2^/d d1–5 or capecitabine 1000 mg/m^2^ bid d1-14)
ATTRACTION-4 (III, double-blind)	Lancet Onco (2022)	NCT02746796 (1:1)	362/362 (64/65)	(523/201)	G/GEJ	Asian (100)	Nivolumab + chemotherapy (oxaliplatin 130 mg/m² d1 + S-1–40 mg/m² or capecitabine 1000 mg/m² bid d1-14)	Placebo + chemotherapy (oxaliplatin 130 mg/m² d1 + S-1–40 mg/m² or capecitabine 1000 mg/m², bid d1-14)
CheckMate 648 (III, open-label)	NEJM (2022)	NCT03143153 (1:1:1)*	321/324 (64/64)	(528/117)	ESCC	Asian (70.4) White (26.6)	Nivolumab + chemotherapy (5-fluorouracil 800 mg/m² on d1-5 + cisplatin 80 mg/m² on d1)	Placebo + chemotherapy (5-fluorouracil 800 mg/m² on d1-5 + cisplatin 80 mg/m² on d1)
CheckMate 649 (III, open-label)	Lancet (2021)	NCT02872116 (1:1:1)*	789/792 (62/61)	(1100/481)	GC/GEJ/EAC	Asian (24) White (69)	Nivolumab+ chemotherapy (capecitabine + oxaliplatin q3w or leucovorin + fluorouracil + oxaliplatin q2w)	Chemotherapy (capecitabine + oxaliplatin q3w or leucovorin + fluorouracil + oxaliplatin q2w)
ORIENT-15 (III, double blind)	BMJ (2022)	NCT03748134 (1:1)	327/332 (63/63)	(567/92)	ESCC	Asian (97) White (1.8)	Sintilimab + chemotherapy (cisplatin 75mg/m^2^ + paclitaxel 175 mg/m^2^ or 5-fluorouracil)	Chemotherapy (cisplatin 75mg/m^2^ + paclitaxel 175 mg/m^2^ or 5-fluorouracil)
ORIENT-16 (III, double-blind)	ESMO (2021)	NCT03745170 (1:1)	327/323	NR	G/GEJ	NR	Sintilimab + chemotherapy (oxaliplatin 130mg/m^2^ + capecitabine 1000 mg/m^2^ bid d1–14 q3w)	Placebo + chemotherapy (oxaliplatin 130mg/m^2^ + capecitabine 1000 mg/m^2^ bid d1–14 q3w)
E SCORT-1st (III, double-blind)	JAMA (2022)	NCT03691090 (1:1)	298/298 (62/62)	(523/73)	ESCC	Asian (100)	Camrelizumab + chemotherapy (paclitaxel 175 mg/m^2^ + cisplatin 75 mg/m^2^)	Placebo + chemotherapy (paclitaxel 175 mg/m^2^ + cisplatin 75 mg/m^2^)
JUPITER-06 (III, double-blind)	Cancer Cell (2022)	NCT03829969 (1:1)	257/257 (63/62)	(437/77)	ESCC	Asian (100)	Toripalimab + chemotherapy (paclitaxel 175 mg/m^2^ + cisplatin 75 mg/m^2^ q3w)	Placebo + chemotherapy (paclitaxel 175 mg/m^2^ + cisplatin 75 mg/m^2^ q3w)

*indicates that these studies have three arms, but data inclusion and analysis use only two of the required; NR, not reported by the included studies.

### 3.2 Risk of bias assessment

The overall risk of 9 RCTs was low with the RoB 2 tool. The quality findings revealed that (1) there was a low RoB in the missing outcome data and the measurement of outcomes; (2) the randomization procedure in one study (CheckMate 648) was uncertain; and (3) the deviation from the intended intervention in one study (CheckMate 649) was also uncertain. Additionally, because the ORIENT-16 study was based on a conference article, the data were incomplete, and the selection of reported outcomes was uncertain. Six RCTs were assessed as low risk, and three as some concerns (**Figure 2**).

**Figure 2:**
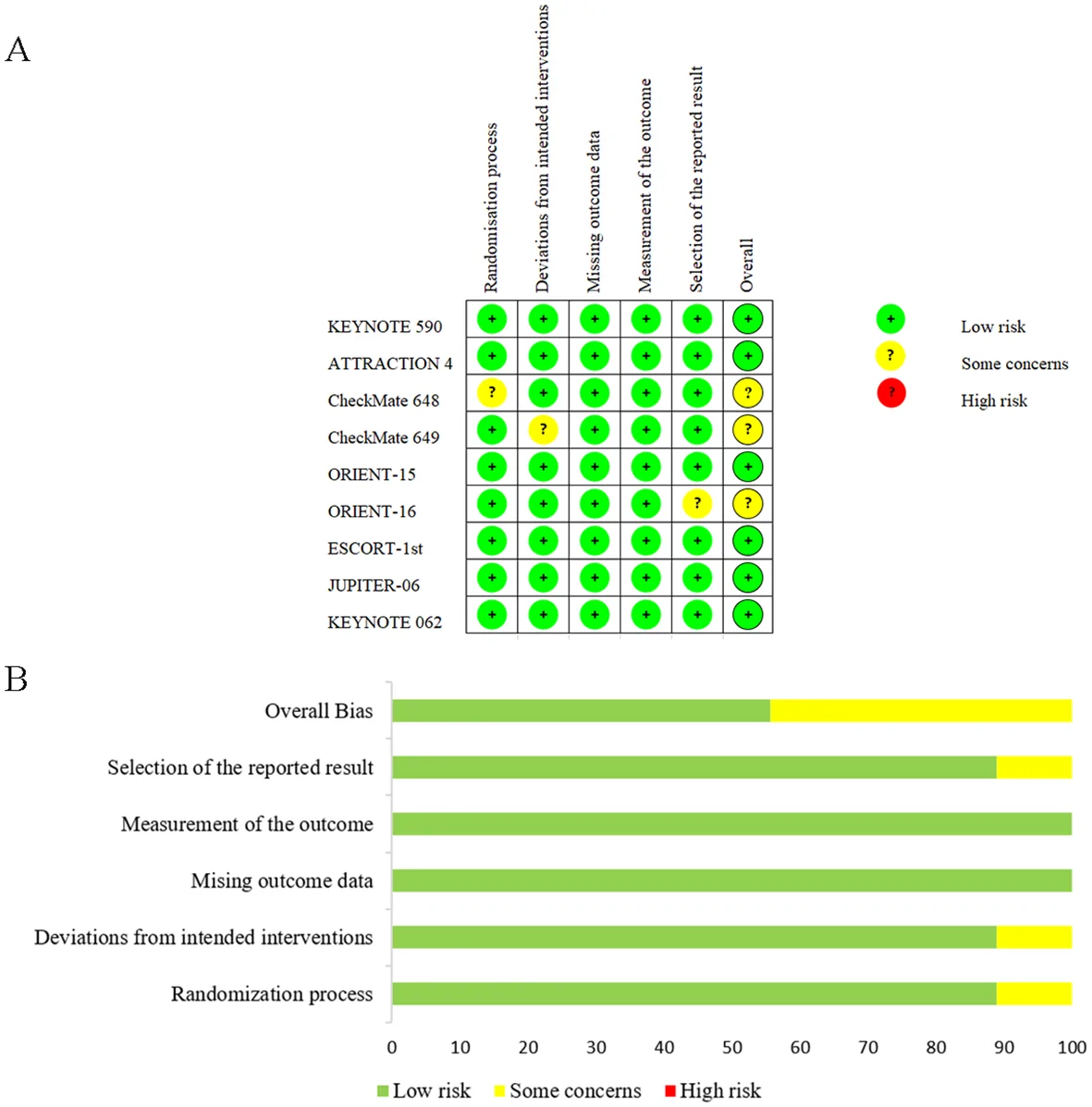
Rob2 risk assessment chart. **(A)** Risk of bias of studies with intention-to-treat with the Cochrane RoB2 tool. **(B)** The summary of risk of bias with included studies. (Green indicates low risks, yellow indicates some concerns, and red indicates high risk; RoB assessment evaluated the intention-to-treat (ITT) effect).

### 3.3 Efficacy

#### 3.3.1 Overall survival

The overall results showed that chemotherapy plus immunotherapy significantly improve OS compared to chemotherapy alone. OS was extended by 2.21 months in comparison to the chemotherapy group (n=5461, MD = 2.21, 95% CI: 1.39-3.03, P<0.00001; **Figure 3A1**) with negligible heterogeneity (I^2^ = 0%, P = 0.59). Compared with chemotherapy alone, PD-1 inhibitors plus chemotherapy were associated with a significantly lower mortality rate (n=6118, HR = 0.75, 95% CI: 0.69-0.81; **Figure 3A2**) with negligible heterogeneity (I^2^ = 35%, P = 0.15). In comparison to chemotherapy, sinti-chemo exhibited the largest OS benefit among all the PD-1 inhibitors. (OS benefit prolonged by 5.5 months, 95% CI: 1.63-9.37, P = 0.005), followed by camre-chemo (OS benefit prolonged by 2.9 months, 95% CI: 0.37-5.43, P = 0.02; **Supplementary Figure 4A**; **Supplementary Table 2**).

**Figure 3:**
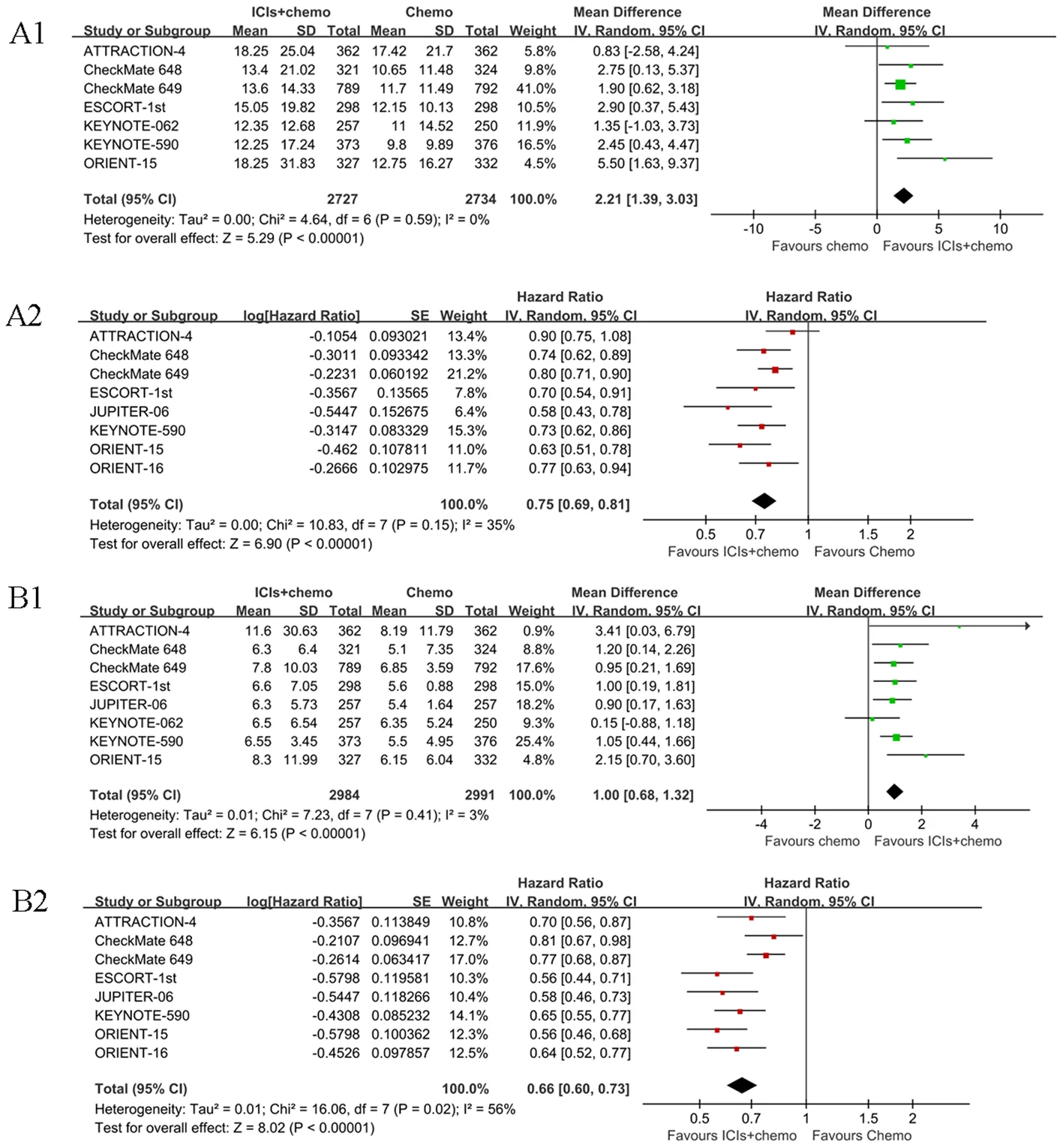
Forest plot of the efficacy of PD-1 inhibitors in AEC/GEJC/AGC patients. **(A1)** OS evaluated with MDs; **(A2)** OS evaluated with HRs; **(B1)** PFS evaluated with MDs; **(B2)** PFS evaluated with HRs (AEC, advanced esophageal cancer; GEJC, gastroesophageal junction carcinoma; AGC, advanced gastric cancer; OS, overall survival; PFS, progress-free survival; MDs, mean deviations; HRs, hazard ratios).

In a subgroup analysis based on PD-L1 status, the PD-1 combined with the chemotherapy group was a highly efficacious and viable treatment option compared to the chemotherapy group in both the PD-L1 CPS positive (HR = 0.67, 95% CI: 0.60-0.74, P<0.00001; **Figure 4A**) and TPS positive (HR = 0.63, 95% CI: 0.55-0.71, P<0.00001; **Figure 4C**).

**Figure 4:**
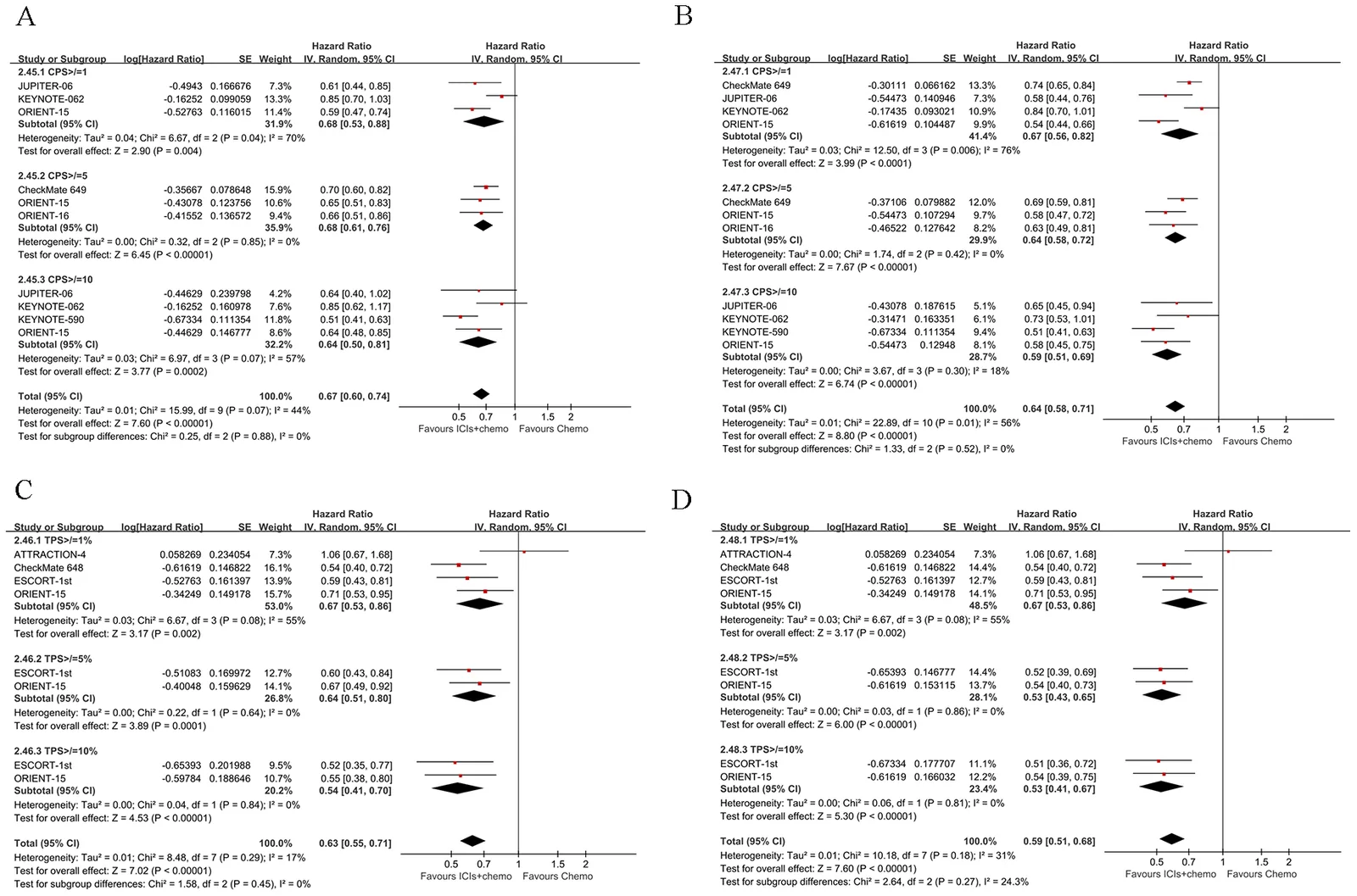
Subgroup analysis of efficacy according to PD-L1 status. **(A)** OS subgroup analysis by PD-1 CPS score. **(B)** PFS subgroup analysis by PD-1 CPS core. **(C)** OS subgroup analysis by PD-L1 TPS score. **(D)** PFS subgroup analysis by PD-L1 TPS score. (CPS, Combined PositiveScore; TPS, Tumor ProportionScore; Of note, the PD-L1 subgroup analyses as exploratory).

#### 3.3.2 Progression-free survival

Analyzing 5975 patients from 8 RCT studies, PD-1 combined with chemotherapy noticeably prolonged PFS compared to chemotherapy, with an overall PFS extension of 1.00 months (95% CI: 0.68-1.32, P<0.00001; **Figure 3B1**). All included studies had low heterogeneity (I^2^ = 3%, P = 0.41). PD-1 inhibitors in combination with chemotherapy reduced the incidence of disease progression by 34% compared to chemotherapy alone (n=6118, HR = 0.66, 95% CI: 0.60-0.73; **Figure 3B2**). In a pooled analysis by PD-1 type, sintilimab in combination with chemotherapy outperformed placebo plus chemotherapy in terms of PFS, with PFS extended by 2.15 months (95% CI: 0.70-3.60, P = 0.004; **Supplementary Figure 4B**; **Supplementary Table 2**).

In a subgroup analysis of PFS based on PD-L1 status, the benefit of PFS in the PD-1 combination chemotherapy group was found in both the PD-L1 CPS positive (HR = 0.64, 95% CI: 0.58-0.71, P<0.00001; **Figure 4B**) and TPS positive (HR = 0.59, 95% CI: 0.51-0.68, P<0.00001; **Figure 4D**), in which the PD-L1 subgroup analyses were performed for exploratory purposes only.

#### 3.3.3 Objective response rate

The results from 9 RCTs with 6625 patients discovered that immunotherapy plus chemotherapy improved the ORR of patients compared to chemotherapy, with an overall OR of 1.77 (95% CI: 1.55-2.03, P<0.00001; **Supplementary Figure 1A**) while there may be moderate heterogeneity (I^2^ = 44%, P = 0.07).

Toripalimab combined with chemotherapy ranked first (OR = 2.07, 95% CI: 1.44-2.97, P<0.0001; **Supplementary Table 2**), pembrolizumab combined with chemotherapy ranked second (OR = 1.81, 95% CI: 1.44- 2.28, P<0.00001), and nivolumab plus chemotherapy ranked third (OR = 1.77, 95% CI: 1.36-2.31, P<0.0001) compared with chemotherapy in terms of ORR improvement. Only sintilimab in combination with chemotherapy did not differ significantly from chemotherapy (OR = 1.75, 95% CI: 0.99-3.08, P = 0.05; **Supplementary Table 2**).

#### 3.3.4 Duration of response and disease control rate

ICIs combined with chemotherapy outperformed chemotherapy in the duration of response (DOR) by 1.99 months in seven studies involving 4275 patients (P<0.00001; **Supplementary Figure 1B**). Subgroup analysis revealed that sintilimab in combination with chemotherapy provided the greatest DOR benefit (MD = 4.00, 95% CI: 0.60-7.40, P = 0.02; **Supplementary Table 2**).

The results from 8 RCTs with 5605 patients discovered that immunotherapy plus chemotherapy improved the disease control rate (DCR) of patients compared to chemotherapy, with an overall OR of 1.37 (95% CI: 1.18-1.59, P<0.0001; **Supplementary Figure 1C**). Subgroup analysis revealed that toripalimab in combination with chemotherapy provided the greatest DCR benefit (OR = 1.78, 95% CI: 1.08-2.96, P = 0.02; **Supplementary Table 2**).

### 3.4 Safety and toxicity

#### 3.4.1 TRAE

The results from 9 RCTs with 6612 patients indicated that PD-1 combination chemotherapy increased TRAE (OR = 1.99, 95% CI: 1.54-2.58, P<0.00001; **Table 2**; **Supplementary Figure 2A**) with the low heterogeneity (I^2^ = 6%, P = 0.39) compared to chemotherapy. Pembrolizumab, sintilimab, and toripalimab combination chemotherapy did not increase TRAE when compared to placebo combination with chemotherapy (P>0.05); however, nivolumab (OR = 2.13, 95% CI: 1.55-2.92, P<0.00001) and camrelizumab (OR = 5.14, 95% CI: 1.12-23.66, P = 0.04; **Table 2**) combination chemotherapy increased TRAE.

**Table 2:** Subgroup analysis of safety on PD-1 types.

AE types	Statistical results	Sintilimab+chemo	Nivolumab+chemo	Pembrolizumab+chemo	Camrelizumab+chemo	Toripalimab+chemo
TRAE	No of patients	327	1472	623	298	257
	Odds Ratio(95%CI)	0.98 (0.31, 3.09)	2.13 (1.55, 2.92)	1.61(0.87-2.97)	5.14 (1.12, 23.66)	1.00 (0.14, 7.15)
	Z value	0.03	4.67	1.51	2.10	0.00
	P (effect)	0.98	<0.00001*	0.13	0.04*	1.00
	Heterogeneity I^2^(%); P (het)	NA	18%; 0.30	0%; 0.40	NA	NA
Grade 3–5 TRAE	No of patients	654	1472	623	298	257
	Odds Ratio (95%CI)	1.31 (1.05, 1.63)	1.70 (1.42, 2.02)	1.24(0.94-1.64)	0.84 (0.60, 1.17)	1.17 (0.79, 1.71)
	Z value	2.43	5.91	1.55	1.03	0.78
	P (effect)	0.02*	<0.00001*	0.12	0.30	0.43
	Heterogeneity I^2^(%);P (het)	0%; 0.65	26%; 0.26	0%; 0.85	NA	NA
SAE	No of patients	327	1472	NA	287	257
	Odds Ratio (95%CI)	1.47 (1.03, 2.11)	1.95 (1.61, 2.37)	NA	1.51 (1.05, 2.18)	1.40 (0.97, 2.03)
	Z value	2.11	6.75	NA	2.20	1.79
	P (effect)	0.03*	<0.00001*	NA	0.03*	0.07
	Heterogeneity I^2^(%);P (het)	NA	0%; 0.67	NA	NA	NA
AE-related treatment discontinuation	No of patients	327	1472	623	298	257
	Odds Ratio (95%CI)	1.86 (1.22, 2.84)	1.92 (1.60, 2.30)	1.46(1.10-1.92)	2.65 (1.86, 3.76)	1.99 (1.06, 3.75)
	Z value	2.89	7.03	2.66	5.44	2.13
	P (effect)	0.004*	<0.00001*	0.008*	<0.00001*	0.03*
	Heterogeneity I^2^(%);P (het)	NA	0%; 0.39	5%; 0.30	NA	NA
AE-related deaths	No of patients	327	1472	623	298	257
	Odds Ratio (95%CI)	1.54 (0.54, 4.37)	1.45 (0.47, 4.55)	0.82(0.46-1.44)	0.81 (0.33, 1.99)	0.33 (0.03, 3.20)
	Z value	0.81	0.65	0.70	0.45	0.96
	P (effect)	0.42	0.52	0.48	0.65	0.34
	Heterogeneity I^2^(%);P (het)	NA	59%; 0.08	9%; 0.29	NA	NA

*p ≤ 0.05, indicates that there has a statistical difference compared with chemotherapy; P (effect), P-value in the effect test; P (het), P-value in the heterogeneity test. Chemo, chemotherapy; TRAE, treatment-related adverse events; SAE, serious adverse events; NA, not applicable.

#### 3.4.2 Grade 3–5 TRAE

When compared to placebo plus chemotherapy, PD-1 combination chemotherapy increased the number of Grade 3–5 TRAE (OR = 1.34, 95% CI: 1.13-1.59, P = 0.007; **Table 2**; **Supplementary Figure 2B**) with a high degree of heterogeneity (I^2^ = 62%, P = 0.007). Subgroup analysis by PD-1 type revealed that pembrolizumab, camrelizumab, and toripalimab plus chemotherapy did not increase the incidence of Grade 3–5 TRAE when compared to chemotherapy (all the P>0.05; **Table 2**). However, nivolumab (OR = 1.70, 95% CI: 1.41-2.02, P<0.00001) and sintilimab (OR = 1.31, 95% CI: 1.05-1.63, P = 0.02, respectively; **Table 2**) increased the risk of Grade 3–5 TRAE by 0.7 and 0.31 times.

#### 3.4.3 SAE and AE leading to discontinuation or death

PD-1 combination chemotherapy, as compared to chemotherapy, increased the incidence of SAE (OR = 1.71, 95% CI: 1.48-1.97, P<0.00001; **Supplementary Figure 2C**) and discontinuation due to adverse effects (OR = 1.87, 95% CI: 1.58-2.20, P<0.00001; **Table 2**). However, there was no significant difference (OR = 1.07, 95% CI: 0.67-1.69, P = 0.78; **Table 2**) between the two groups in terms of death due to AE.

#### 3.4.4 Based on types of TRAE and IRAE

The most common treatment-related AE are anemia, nausea, decreased neutrophil count, and decreased appetite (**Figure 5A**), while the most common immune-related AE are rash, hypothyroidism, and pruritus (**Figure 5C**; **Supplementary Table 3**). Camre-chemo has the highest rate of anemia (79.8%, **Figure 5B**; **Supplementary Table 3**), while tori-chemo has the highest rate of rash (21.8%, **Figure 5D**; **Supplementary Table 3**). Sint-chemo has the highest rate of immune-related hypothyroidism (14.7%) and pem-chemo has the highest rate of immune-related pneumonitis (4.5%, **Figure 5D**; **Supplementary Table 3**).

**Figure 5:**
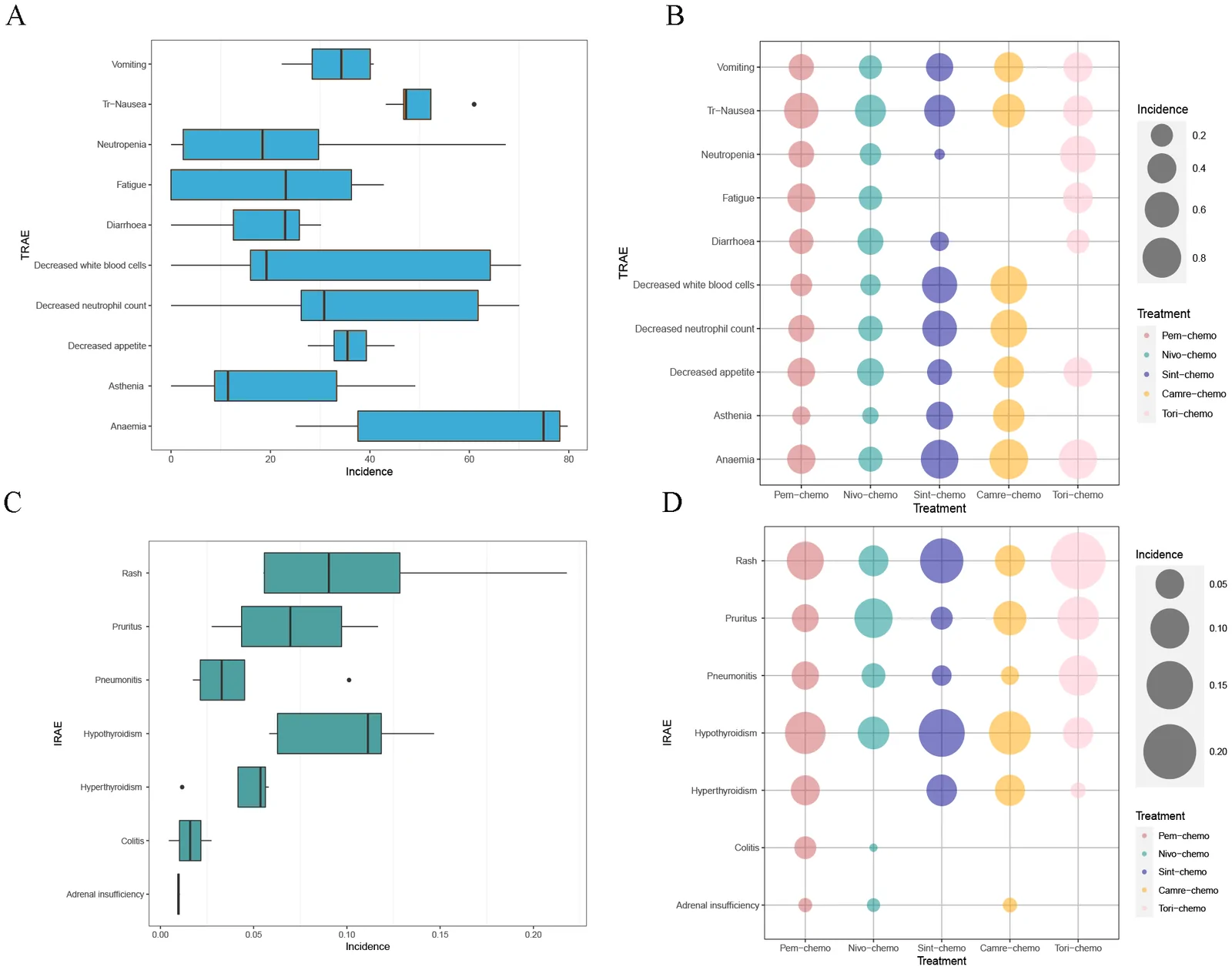
Incidence of treatment-related and immune-mediated adverse events. **(A)** Boxplot of the incidence of treatment-related adverse events. **(B)** Bubble plot of the incidence of treatment-related adverse events. **(C)** Boxplot of the incidence of immune-related adverse events. **(D)** Bubble plot of the incidence of immune-related adverse events. Pem, pembrolizumab; Nivo, nivolumab; Sint, sintilimab; Camre, camrelizumab; Tori, toripalimab; chemo, chemotherapy. (**Supplementary Table 3** provides exact numerical values for **Figure 5**).

### 3.5 Sensitivity analysis

The included RCTs were divided into subgroups based on their characteristics. **Supplementary Table 4** shows the robust results OS, PFS, ORR, and TRAE in different subgroups. Sensitivity analysis of the efficacy data suggested that the type of drug may be responsible for the heterogeneity in PFS. What is more, the cause of ORR heterogeneity may be connected to sample size and bias risk. Sensitivity revealed that the source of heterogeneity in TRAE was related to drug type and sample size in terms of safety data.

### 3.6 Publication biases

The funnel plot illustrates the possible publication bias of the included RCTs (**Supplementary Figure 3**). The results show that one RCT deviated from the midline in each of the analyses for OS and PFS and there was no apparent publication bias for ORR and SAE, but one RCT deviated from the midline in TRAE.

### 3.7 Moderate evidence strength

The original evidence strength of this meta-analysis was defined as high because all included studies were RCTs (⊕⊕⊕⊕) with the GRADE approach. However, publication bias was found in OS, PFS, and DOR data that lowered the evidence strength to moderate(⊕⊕⊕◯). Furthermore, significant publication bias reduced the evidence strength of TRAE. Because of the inconsistency (I^2^ = 44%), the evidence strength of ORR data was reduced to moderate(⊕⊕⊕◯). The IRAE evidence strength was very low due to significant inconsistency (I^2^ = 92%) and publication bias. As a result, the overall strength of the evidence was rated as moderate (**Supplementary Table 5**) that indicate immunotherapy combined with chemotherapy may be considered as a viable option.

## 4 Discussion

### 4.1 Main findings

To our knowledge, this is the first PRISMA-compliant meta-analysis to compare the efficacy and safety of various immune-chemo combinations vs chemotherapy monotherapy as the first-line therapy for advanced gastroesophageal cancer. The latest findings provide evidence for clinical practice with a well-planned and thorough synthesis, including the following:

1. PD-1 and chemotherapy combinations outperformed standard chemotherapy regarding OS, PFS, and ORR.
2. PD-1 plus chemotherapy increased TRAE, Grade 3–5 TRAE, andSAE compared to standard chemotherapy. However, there was no difference in AE-related deaths.
3. Sinti-chemo had the best OS, PFS, and DOR, in addition to the lower incidence of treatment-related adverse events (no significant difference compared to standard chemotherapy) compared to other PD-1 therapies.
4. Torip-chemo provided the best ORR and DCR and had the lower incidence ofSAE (no significant difference compared to standard chemotherapy).
5. According to subgroup analyses based on PD-L1 status, OS and PFS benefit was observed in all groups with PD-L1 CPS positive and TPS positive.
6. Sint-chemo ranked first for OS, PFS, and DOR and minimal for TRAE, the second least forSAE among all immuno-chemo combinations, demonstrating a good compromise between effectiveness and safety.

### 4.2 Reliability of this study

Based on 9 RCTs with 6625 participants, this comprehensive study is the first PRISMA-compliant meta-analysis to evaluate the effectiveness and safety of immune-chemo versus chemotherapy in treating patients with gastroesophageal adenocarcinoma. In terms of efficacy, immune-chemo resulted in a 2.21-month increase in OS over single-agent chemotherapy (p < 0.00001) and reduced mortality rate by 25% (p < 0.00001); in terms of safety, immune-chemo resulted in an increase in TRAE over single-agent chemotherapy (OR = 1.99, p < 0.00001). The evidence for immune-chemotherapy with robust clinical efficacy was supported by subgroup and sensitivity analyses of various parameters, including sample size, year of publication, PD-1 type, and race. It was also suggested that the source of heterogeneity may be related to drug type, which prompted additional subgroup analyses.

### 4.3 The novelty of this study

The efficacy and safety of immune-chemotherapy against chemotherapy in the treatment of gastroesophageal cancer were compared in this updated meta-analysis. With five phase III clinical studies published online in 2022, this study was the first research to compare first-line immune-chemo treatments for gastroesophageal cancer and investigate which agent was the best. More importantly, the consistency and robustness of the findings of this study were tested using appropriate basic statistical procedures, including publication bias analysis, sensitivity analysis, and subgroup analysis. The GRADE approach was used to evaluate the overall strength of the evidence, and the ROB2 tool was utilized to evaluate the quality of the evidence in the included RCTs.

### 4.4 Results interpretation

Previous studies evaluated the efficacy of PD-1 versus chemotherapy for patients with advanced gastroesophageal cancer (25, 26). Our findings that PD-1 combined with chemotherapy significantly improves efficacy extended previous findings. The mechanism may be related to cytotoxic drugs increasing tumor cell immunogenicity. Chemotherapy drugs could cause immunogenic cell death (ICD), which causes an immune response to dead-cell antigens (27). The production of ICD might improve the therapeutic benefits of ICIs by enhancing immune response. A promising strategy for enhancing the clinical results of cancer patients is the combination of ICD-inducing chemotherapeutics with immunotherapy (28). The chemotherapeutic agents in this study were mainly platinum-based chemotherapy, while previous studies of platinum-based chemotherapy on immunomodulation can be used to boost the susceptibility of tumor cells to PD-1 or PD-L1 inhibitors and upregulate PD-L1 expression in tumor tissue and have a detrimental immunomodulatory effect (29–31).

Subgroup analysis of patients discovered that PD-L1 status could be used to assess the efficacy of immunotherapy. The results showed that there was OS and PFS benefit in all groups with PD-L1 CPS positive and TPS positive. Previous research has demonstrated that PD-L1 status is a significant factor related to prognosis (32–34). Researchers discovered a consistent benefit of immune checkpoint inhibitors in lowering the risk of death in ESCC patients in a meta-analysis, and that survival benefit was associated with PD-L1 CPS status (35). However, there are some debates about which scoring method is a better predictor. The efficacy of the CPS score and TPS score for predicting immunotherapy response is consistent in a study of non-small cell lung cancer (36). A study on gastric cancer, however, found that CPS was a more useful assessment method than TPS as a prognostic biomarker (21). There may be some differences between the PD-L1 CPS score and the TPS score in predicting PD-1 inhibitors efficacy in real-world clinical studies. Further research is required to determine the relationship between PD-L1 expression status and immune checkpoint inhibitor effectiveness.

Although CheckMate 648, CheckMate 649, and ATTRACTION-4 selected the same PD-1 as the experimental group, their efficacies varied significantly, the different pathological types of included patients would be the possible explanations. Patients included in CheckMate 648 were all squamous carcinomas, whereas patients in the latter two RCTs suffered from both squamous and adenocarcinomas. Previous studies discovered that ESCC and EAC contain different tumor stem cells that display a variety of cell surface markers and have distinct cell origins (37), which suggests that different tumor pathology types have different driver genes and impacts on the efficacy of immunotherapy. A study that evaluated the molecular characteristics of three different types of gastroesophageal cancer found that ESCC had higher PD-L1 expression than EAC and GAC (38) and immunotherapy response was correlated with PD-L1 expression level (32, 39, 40). Therefore, further research on different types of pathology and treatment effects is needed.

Sintilimab trials (e.g., ORIENT-15) were predominantly composed of ESCC patients, a subtype that has demonstrated robust and consistent sensitivity to single-agent or combination PD-1 inhibition in several landmark trials. Conversely, the pivotal trials for nivolumab and pembrolizumab (e.g., CheckMate 649, KEYNOTE-062) enrolled larger proportions of, or exclusively, patients with adenocarcinoma/GEJC. Of note is that this predominance of the highly immune-responsive ESCC histology in the sintilimab pooled data may act as a confounding factor, potentially biasing the apparent “superiority” or highly favorable relative efficacy of sintilimab observed in cross-trial comparisons. These issues will be specifically addressed in our future large-scale network meta-analysis studies.

It has been shown that ORR, DCR, PFS, and other efficacy indicators cannot replace OS as the endpoint of PD-1/PD-L1 inhibitor therapy (41). Therefore, while tori-chemo had the best ORR and DCR, we still consider that sint-chemo has the best efficacy because of its longest OS benefit. The PD-L1-based subgroup analyses were exploratory in nature because CPS and TPS are not interchangeable. Thus, pooling these data may represent a significant source of measurement bias. Previous research has looked into the connection between clinical characteristics and PD-1 efficacy. Age may be a factor since the survival benefit of CTLA-4 inhibitors is lower in older patients (42). Age-related benefits may vary between different types of ICI. Another factor is the type of platinum, meta-analysis showed that PD-1 inhibitors combined with oxaliplatin prolonged PFS better than PD-1 combined with cisplatin (43). This may explain the efficacy differences between ATTRACTION-4 and CheckMate 648. What’s more, gender is also a factor in PD-1 efficacy, with men benefiting more from PD-1 treatment than women (44). Future research is required to investigate these clinical characteristics.

### 4.5 Hypothesis-generating considerations

This study found that sintilimab in combination with chemotherapy did not increase the incidence of TRAE compared with chemotherapy. The possible reason may be its drug structure. To prevent Fab segment swapping, all of the IgG4 subtypes of PD-1 that are currently on the market had their Fab segments modified. The Fc segment regulates immunological responses, such as complement-dependent cytotoxicity (CDC), antibody-dependent cell phagocytosis (ADCP), antibody-dependent cell-mediated cytotoxicity (ADCC), and the half-life of antibodies in serum. Different immune checkpoint inhibitors have different crystal structures of Fab fragment complexes of therapeutic antibodies (45). Sintilimab has little to no affinity for Fc receptors for IgG (FcgR), suggesting that it has little effect on complement- or antibody-dependent cytotoxicity (ADCR) (46). Its unique structure may be responsible for improved efficacy and safety, which provides a research direction for future drug development and manufacturing.

Hypothyroidism is a common immune adverse effect during PD-1 inhibitors treatment. While PD-1 inhibitors activate host T cells against malignant tumors, they also attack normal cells. Normal thyroid cells are susceptible to attack by cytotoxic T lymphocytes. Due to the better affinity and lower dissociation rate, sintilimab release more efficiently into the blood (47), which may lead to sinti-chemo’s high incidence rate of hypothyroid. Similarly, the T-cell response caused by PD-1 inhibitors can lead to a range of side effects, including interstitial pneumonia (48). It has been reported that interstitial pneumonia is related to the patient’s lung disease condition and history of chest radiation (49). However, there is no explicit explanation of the specific pneumonitis related to Pem-chemo and further studies are pending. These molecular mechanisms should be considered potential explanations warranting further investigation rather than established causal mechanisms underlying the observed safety profiles.

### 4.6 Implications

This study synthesizes data from all currently eligible clinical trials to provide updated evidence for treatment selection among multiple options. In terms of both efficacy and safety, sintilimab in combination with chemotherapy seems to be the best treatment choice for patients with advanced gastroesophageal cancer. The addition of PD-1 exacerbates toxicity but does not increase the risk of treatment-related death compared with standard chemotherapy. Sintilimab exhibited the lower incidence of treatment-related AEs, whereas toripalimab also had the lower incidence of SAEs. However, it should be explicitly state that while sintilimab plus chemotherapy has a comparable rate of any-grade TRAEs relative to chemotherapy alone, it carries a statistically significant increased risk of severe toxicities, specifically Grade 3–5 TRAEs (OR 1.31, P = 0.02) and serious adverse events (SAEs, OR 1.47, P = 0.03). These findings may guide immunotherapy selection in patients with poor performance status and inform whether immunotherapy plus chemotherapy should be incorporated into standard care for advanced gastroesophageal cancer.

However, further head-to-head comparison trials are still needed, as well as clinical research on PD-1 in combination with targeted therapies or other immune checkpoint inhibitors such as PD-L1 or CTLA-4. While our meta-analysis focused on the RCTs defined by our pre-specified search protocol, the validity of our findings is reinforced by emerging data from 2024 and 2025. Notably, the significant survival gains observed in the RATIONALE-305 and 306 trials corroborate the higher efficacious of PD-1 combinations in this study (50, 51). Furthermore, the FDA approval of Tevimbra in March 2025 represents a significant milestone in first-line ESCC treatment. Future research should prioritize head-to-head comparisons of structurally optimized agents, such as sintilimab and tislelizumab. Meanwhile, investigations into Fc-segment modifications and the clinical utility of the Tumor Area Positivity score may further refine personalized therapy.

### 4.7 Limitations

There are still some limitations to this study. First, compared to head-to-head RCTs, this meta-analysis could not have as much validating power. Second, fewer RCTs were included when analyzing the impact of PD-L1 status on efficacy. A small sample size could affect the evaluation of the association between PD-L1 expression and effectiveness. A further limitation of this study is that comparisons among different PD-1 inhibitors were based on indirect subgroup analyses. Owing to the absence of head-to-head randomized controlled trials and a formal network meta-analysis, definitive conclusions regarding the numerically more favorable outcomes of sintilimab over other PD-1 inhibitors from indirect comparisons cannot be firmly established. It should be noted that indirect comparisons on cross-trials may be confounded by differences in eligibility criteria, PD-L1 assays/cut-offs, chemotherapy backbones. Future prospective, randomized, comparative studies are warranted to validate these preliminary efficacy findings. Third, the intervention arm only included PD-1 in combination with chemotherapy, there was neither PD-L1/CTLA-4 in combination with chemotherapy nor immune checkpoint inhibitor in combination with targeted therapy, so new clinical trials of immune checkpoint inhibitor combination with targeted therapy deserve further study. Fourth, a formal meta-regression by PD-L1 cut-offs would lack sufficient statistical power and could yield unreliable estimates due to the limited number of included trials and the highly fragmented nature of the reported cut-offs (e.g., CPS ≥ 1, ≥ 5, ≥ 10) and scoring systems across studies. Fifth, the sample size from 9 RCTs with 5 different PD-1 inhibitors was too small to conduct any ranking analysis. The results of the ranking analysis based on current data would be unstable and unreliable. Sixth, recent phase III trials (including RATIONALE-305 and RATIONALE-306) (50, 51) were excluded from the quantitative synthesis due to publication/reporting after our pre-defined search cut-off. Thus, a comprehensive network meta-analysis will be conducted to obtain more evidence including ranking analysis, more potential confounding effects (such as tumor histology) on more reliable RCTs when more high-quality RCTs with more drugs and adequate information are available in future.

## 5 Conclusions

Findings from this comprehensive study confirm that immunotherapy combined with chemotherapy is a more efficacious first-line strategy than conventional chemotherapy alone for advanced unresectable GEC. Notably, sintilimab combined with chemotherapy demonstrated greater OS and PFS benefits than other PD-1 inhibitor-chemotherapy regimens without its statistically significant increase in overall TRAEs, though Grade 3–5 TRAE was increased base on indirect comparisons. The moderate evidence indicated that PD-1 combined with chemotherapy may be a viable option for advanced unresectable GEC across PD-L1 expression subgroups.

## Data Availability

The original contributions presented in the study are included in the article/**Supplementary Material**. Further inquiries can be directed to the corresponding authors.
