# Supplementary material for: Efficacy and safety of PD-1 inhibitors plus chemotherapy for advanced unresectable gastroesophageal cancer: a systematic review and meta-analysis of phase III randomized controlled trials
Source: Front Oncol. 2026 Sep 22;16:1838929. doi: 10.3389/fonc.2026.1838929 (PMC13638392; doi:10.3389/fonc.2026.1838929)
Supplement: Supplementary file 1 [file SupplementaryFile1.docx]

Supplemental Content

[Figure S1. Forest plot of the efficacy of PD-1 inhibitors in AEC/ GEJC /AGC patients.	2](#_Toc131801323)

[Figure S2. Forest plot of the safety of PD-1 inhibitors in AEC/ GEJC /AGC patients.	3](#_Toc131801324)

[Figure S3. Funnel plot of the main outcomes.	4](#_Toc131801325)

[Figure S4. Subgroup analysis of efficacy according to type of PD-1 inhibitors. (A) OS; (B) PFS.	5](#_Toc131801326)

[Table S1. PRISMA 2020 Checklist.	6](#_Toc131801327)

[Table S2. Subgroup analysis of the efficacy.	10](#_Toc131801328)

[(1) OS subgroup analysis	10](#_Toc131801329)

[(2) PFS subgroup analysis	11](#_Toc131801330)

[(3) DOR、ORR、DCR subgroup analysis by PD-1 types	12](#_Toc131801331)

[Table S3. Incidence of treatment-related and immune-mediated adverse events.	14](#_Toc131801332)

[(1) Treatment-related adverse events.	14](#_Toc131801333)

[(2) Immune-mediated adverse events.	14](#_Toc131801334)

[Table S4. Sensitivity analyses of efficacy and safety.	15](#_Toc131801335)

[(1) OS	15](#_Toc131801336)

[(2) PFS	15](#_Toc131801337)

[(3) ORR	16](#_Toc131801338)

[(4) TRAE	17](#_Toc131801339)

[Table S5. GRADE approach.	19](#_Toc131801340)

[Supplementary S1. Search strategies.	22](#_Toc131801341)

[(1) PubMed	22](#_Toc131801342)

[(2) Web of Science	22](#_Toc131801343)

[(3) Cochrane Library	23](#_Toc131801344)

[(4) Embase	24](#_Toc131801345)

# **Figure S1. Forest plot of the efficacy of PD-1 inhibitors in AEC/ GEJC /AGC patients.**

(A) ORR; (B) DOR; (C) DCR (AEC: advanced esophageal cancer; GEJC: gastroesophageal junction carcinoma; AGC: advanced gastric cancer; ORR: objective response rate; DOR: duration of response; DCR: disease control rate)

(A) ORR

(B) DOR

(C) DCR

# **Figure S2. Forest plot of the safety of PD-1 inhibitors in AEC/ GEJC /AGC patients.**

(A) TRAE; (B) Grade 3-5 TRAE; (C) SAE; (D) IRAE (AEC: advanced esophageal cancer; GEJC: gastroesophageal junction carcinoma; AGC: advanced gastric cancer; TRAE: treatment-related adverse events; SAE: serious adverse events; IRAE: immune-related adverse events)

(A) TRAE

(B) Grade 3-5 TRAE

(C) SAE

(D) IRAE

# **Figure S3. Funnel plot of the main outcomes.**

(A) OS; (B) PFS; (C) ORR; (D) TRAE; (E) SAE (OS: overall survival; PFS: progression-free survival; ORR: objective response rate; TRAE: treatment-related adverse event; SAE: serious adverse event)

(A) OS	(B) PFS

(C) ORR	(D) TRAE

(E) SAE

# **Figure S4. Subgroup analysis of efficacy according to type of PD-1 inhibitors. (A) OS; (B) PFS.**

A

B

# Table S1. PRISMA 2020 Checklist.

|  | **Item #** | **Checklist item** | **Location where item is reported** |
| --- | --- | --- | --- |
| **TITLE** | | |  |
| Title | 1 | Identify the report as a systematic review. | Page 1 |
| **ABSTRACT** | | |  |
| Abstract | 2 | See the PRISMA 2020 for Abstracts checklist. | Page 2, 3 |
| **INTRODUCTION** | | |  |
| Rationale | 3 | Describe the rationale for the review in the context of existing knowledge. | Page 2 |
| Objectives | 4 | Provide an explicit statement of the objective(s) or question(s) the review addresses. | Page 2, 3 |
| **METHODS** | | |  |
| Eligibility criteria | 5 | Specify the inclusion and exclusion criteria for the review and how studies were grouped for the syntheses. | Page 3 |
| Information sources | 6 | Specify all databases, registers, websites, organisations, reference lists and other sources searched or consulted to identify studies. Specify the date when each source was last searched or consulted. | Page 3 |
| Search strategy | 7 | Present the full search strategies for all databases, registers and websites, including any filters and limits used. | Page 3, Supplementary S1 |
| Selection process | 8 | Specify the methods used to decide whether a study met the inclusion criteria of the review, including how many reviewers screened each record and each report retrieved, whether they worked independently, and if applicable, details of automation tools used in the process. | Page 3 |
| Data collection process | 9 | Specify the methods used to collect data from reports, including how many reviewers collected data from each report, whether they worked independently, any processes for obtaining or confirming data from study investigators, and if applicable, details of automation tools used in the process. | Page 3 |
| Data items | 10a | List and define all outcomes for which data were sought. Specify whether all results that were compatible with each outcome domain in each study were sought (e.g. for all measures, time points, analyses), and if not, the methods used to decide which results to collect. | Page 3 |
|  | 10b | List and define all other variables for which data were sought (e.g. participant and intervention characteristics, funding sources). Describe any assumptions made about any missing or unclear information. | Page 3 |
| Study risk of bias assessment | 11 | Specify the methods used to assess risk of bias in the included studies, including details of the tool(s) used, how many reviewers assessed each study and whether they worked independently, and if applicable, details of automation tools used in the process. | Page 3 |
| Effect measures | 12 | Specify for each outcome the effect measure(s) (e.g. risk ratio, mean difference) used in the synthesis or presentation of results. | Page 3, 4 |
| Synthesis methods | 13a | Describe the processes used to decide which studies were eligible for each synthesis (e.g. tabulating the study intervention characteristics and comparing against the planned groups for each synthesis (item #5)). | Figure 1 |
|  | 13b | Describe any methods required to prepare the data for presentation or synthesis, such as handling of missing summary statistics, or data conversions. | Page 4 |
|  | 13c | Describe any methods used to tabulate or visually display results of individual studies and syntheses. | Page 4 |
|  | 13d | Describe any methods used to synthesize results and provide a rationale for the choice(s). If meta-analysis was performed, describe the model(s), method(s) to identify the presence and extent of statistical heterogeneity, and software package(s) used. | Page 4 |
|  | 13e | Describe any methods used to explore possible causes of heterogeneity among study results (e.g. subgroup analysis, meta-regression). | Page 4 |
|  | 13f | Describe any sensitivity analyses conducted to assess robustness of the synthesized results. | Page 4 |
| Reporting bias assessment | 14 | Describe any methods used to assess risk of bias due to missing results in a synthesis (arising from reporting biases). | Page 3 |
| Certainty assessment | 15 | Describe any methods used to assess certainty (or confidence) in the body of evidence for an outcome. | Page 4 |
| **RESULTS** | | |  |
| Study selection | 16a | Describe the results of the search and selection process, from the number of records identified in the search to the number of studies included in the review, ideally using a flow diagram. | Figure 1 |
|  | 16b | Cite studies that might appear to meet the inclusion criteria, but which were excluded, and explain why they were excluded. | Figure 1 |
| Study characteristics | 17 | Cite each included study and present its characteristics. | Table 1 |
| Risk of bias in studies | 18 | Present assessments of risk of bias for each included study. | Figure 2 |
| Results of individual studies | 19 | For all outcomes, present, for each study: (a) summary statistics for each group (where appropriate) and (b) an effect estimate and its precision (e.g. confidence/credible interval), ideally using structured tables or plots. | Table 2; table S2 |
| Results of syntheses | 20a | For each synthesis, briefly summarise the characteristics and risk of bias among contributing studies. | Page 6 |
|  | 20b | Present results of all statistical syntheses conducted. If meta-analysis was done, present for each the summary estimate and its precision (e.g. confidence/credible interval) and measures of statistical heterogeneity. If comparing groups, describe the direction of the effect. | Page 6, 7, 8, 9, 10 |
|  | 20c | Present results of all investigations of possible causes of heterogeneity among study results. | Page 6, 7, 8, 9, 10 |
|  | 20d | Present results of all sensitivity analyses conducted to assess the robustness of the synthesized results. | Page 6, 7, 8, 9, 10 |
| Reporting biases | 21 | Present assessments of risk of bias due to missing results (arising from reporting biases) for each synthesis assessed. | Figure 2 |
| Certainty of evidence | 22 | Present assessments of certainty (or confidence) in the body of evidence for each outcome assessed. | Table S5 |
| **DISCUSSION** | | |  |
| Discussion | 23a | Provide a general interpretation of the results in the context of other evidence. | Page 10, 11 |
|  | 23b | Discuss any limitations of the evidence included in the review. | Page 11 |
|  | 23c | Discuss any limitations of the review processes used. | Page 11 |
|  | 23d | Discuss implications of the results for practice, policy, and future research. | Page 11, 12 |
| **OTHER INFORMATION** | | |  |
| Registration and protocol | 24a | Provide registration information for the review, including register name and registration number, or state that the review was not registered. | PROSPERO registered protocol (CRD42022325401) |
|  | 24b | Indicate where the review protocol can be accessed, or state that a protocol was not prepared. | Page 3 |
|  | 24c | Describe and explain any amendments to information provided at registration or in the protocol. | Not application |
| Support | 25 | Describe sources of financial or non-financial support for the review, and the role of the funders or sponsors in the review. | Page 13 |
| Competing interests | 26 | Declare any competing interests of review authors. | Page 13 |
| Availability of data, code and other materials | 27 | Report which of the following are publicly available and where they can be found: template data collection forms; data extracted from included studies; data used for all analyses; analytic code; any other materials used in the review. | Page 13 |

*From:* Page MJ, McKenzie JE, Bossuyt PM, Boutron I, Hoffmann TC, Mulrow CD, et al. The PRISMA 2020 statement: an updated guideline for reporting systematic reviews. BMJ 2021;372:n71. doi: 10.1136/bmj.n71

For more information, visit: <http://www.prisma-statement.org/>

# Table S2. Subgroup analysis of the efficacy.

(1) OS subgroup analysis by PD-1 types and PD-L1 status. (2) PFS subgroup analysis by PD-1 types and PD-L 1 status. (3) DOR、ORR、DCR subgroup analysis by PD-1 types. (DOR: duration of response; ORR: objective response rate; DCR: disease control rate).

(1) OS subgroup analysis

|  | No of trials | No of patients | Mean Difference(months)  95%CI | Z value | P value | Heterogeneity within subgroups |
| --- | --- | --- | --- | --- | --- | --- |
| Drug |  |  |  |  |  | **I^2^ (%) P value** |
| Sintilimab+chemo | 1 | 327 | 5.50 (1.63, 9.37) | 2.79 | 0.005 | Not applicable |
| Nivolumab+chemo | 3 | 1472 | 1.94 (0.85, 3.03) | 3.49 | 0.0005 | 0% 0.68 |
| Pembrolizumab+chemo | 2 | 630 | 1.99(0.45-3.53) | 2.54 | 0.01 | 0% 0.49 |
| Camrelizumab+chemo | 1 | 298 | 2.90 (0.37, 5.43) | 2.25 | 0.02 | Not applicable |
| Total | 7 | 2727 | 2.21(1.39-3.03) | 5.29 | <0.00001 | 0% 0.59 |
| PD-L1 status |  |  |  |  |  |  |
| PD-L1 CPS>/=1 | 2 | 898 | 2.07(0.82-3.32) | 3.25 | 0.001 | 0% 0.48 |
| PD-L1 CPS>/=10 | 2 | 285 | 3.04(0.26-5.82) | 2.15 | 0.03 | 25% 0.25 |
| PD-L1 TPS>/=1% | 2 | 216 | 4.27 (-1.41, 9.94) | 1.47 | 0.14 | 64% 0.09 |
| Total | 5 | 1399 | 2.74(1.28-4.19) | 3.69 | 0.0002 | 31% 0.19 |

(2) PFS subgroup analysis

| Drug | No of trials | No of patients | Mean Difference(months)  95%CI | Z value | P value | Heterogeneity within subgroups  **I^2^ (%) P value** |
| --- | --- | --- | --- | --- | --- | --- |
|  |  |  |  |  |  |  |
| Sintilimab+chemo | 1 | 327 | 2.15 (0.70, 3.60) | 2.90 | 0.004 | Not applicable |
| Nivolumab+chemo | 3 | 1472 | 1.11 (0.51, 1.71) | 3.62 | 0.0003 | 0% 0.37 |
| Pembrolizumab+chemo | 2 | 630 | 0.70(-0.16-1.56) | 1.59 | 0.11 | 54% 0.14 |
| Camrelizumab+chemo | 1 | 298 | 1.00 (0.19, 1.81) | 2.43 | 0.02 | Not applicable |
| Toripalimab +chemo | 1 | 257 | 0.90 (0.17, 1.63) | 2.42 | 0.02 | Not applicable |
| Total | 8 | 2727 | 1.03(0.64-1.42) | 5.17 | <0.00001 | 16% 0.31 |
| **PD-L1 positive status** |  |  |  |  |  |  |
| PD-L1 CPS>/=1 | 3 | 1099 | 0.83(0.30-1.36) | 3.06 | 0.002 | 15% 0.31 |
| PD-L1 CPS>/=10 | 3 | 489 | 1.70 (1.02, 2.38) | 4.91 | <0.00001 | 2% 0.38 |
| PD-L1 TPS>/=1% | 3 | 382 | 1.52 (0.60, 2.44) | 3.24 | 0.001 | 0% 0.43 |
| Total | 9 | 1970 | 1.25(0.82-1.69) | 5.61 | <0.00001 | 23% 0.23 |

(3) DOR、ORR、DCR subgroup analysis by PD-1 types

|  |  | Sintilimab+chemo | Nivolumab+chemo | Pembrolizumab+chemo | Camrelizumab+chemo | Toripalimab+chemo |
| --- | --- | --- | --- | --- | --- | --- |
| DOR | No of patients | 327 | 1033 | 257 | 298 | 257 |
|  | Mean Difference(months)  95%CI | 4.00 (0.60, 7.40) | 1.95 (0.82, 3.08) | 0.10(-1.88-2.08) | 2.60 (1.08, 4.12) | 2.25 (0.10, 4.40) |
|  | Z value; P value | 2.31; 0.02 | 3.38; 0.0007 | 0.10; 0.92 | 3.35; 0.0008 | 2.05; 0.04 |
|  | Heterogeneity  I^2^(%); P value | Not applicable | 0%; 0.50 | Not applicable | Not applicable | Not applicable |
| ORR | No of patients | 654 | 1472 | 630 | 298 | 257 |
|  | Odds Ratio (95%CI) | 1.75 (0.99, 3.08) | 1.77 (1.36, 2.31) | 1.81(1.44-2.28) | 1.58 (1.12, 2.23) | 2.07 (1.44, 2.97) |
|  | Z value; P value | 1.93; 0.05 | 4.23; <0.0001 | 5.07; <0.00001 | 2.61; 0.009 | 3.95; <0.0001 |
|  | Heterogeneity  I^2^(%); P value | 84%; 0.01 | 65%; 0.06 | 0%; 0.37 | Not applicable | Not applicable |
| DCR | No of patients | 327 | 1286 | 630 | 298 | 257 |
|  | Odds Ratio (95%CI) | 1.71 (1.07, 2.74) | 1.42 (1.14, 1.77) | 1.10(0.82-1.47) | 1.30 (0.76, 2.24) | 1.78 (1.08, 2.96) |
|  | Z value; P value | 2.24; 0.02 | 3.15; 0.002 | 0.65; 0.52 | 0.96; 0.34 | 2.24; 0.02 |
|  | Heterogeneity  I^2^(%); P value | Not applicable | 26%; 0.26 | 17%; 0.29 | Not applicable | Not applicable |

# Table S3. Incidence of treatment-related and immune-mediated adverse events.

(1) Treatment-related adverse events.

| Incidence | Pem-chemo | Nivo-chemo | Sint-chemo | Camre-chemo | Tori-chemo |
| --- | --- | --- | --- | --- | --- |
| Tr-Nausea | 0.610 | 0.473 | 0.468 | 0.523 | 0.432 |
| Decreased appetite | 0.355 | 0.328 | 0.275 | 0.449 | 0.393 |
| Anaemia | 0.376 | 0.251 | 0.749 | 0.798 | 0.782 |
| Fatigue | 0.363 | 0.231 | NA | NA | 0.428 |
| Decreased neutrophil count | 0.308 | 0.262 | 0.618 | 0.700 | NA |
| Vomiting | 0.284 | 0.223 | 0.343 | 0.408 | 0.401 |
| Diarrhoea | 0.258 | 0.302 | 0.125 | NA | 0.230 |
| Neutropenia | 0.297 | 0.184 | 0.024 | NA | 0.673 |
| Decreased white blood cells | 0.192 | 0.160 | 0.642 | 0.704 | NA |
| Asthenia | 0.115 | 0.088 | 0.333 | 0.491 | NA |

(2) Immune-mediated adverse events.

| Incidence | Pem-chemo | Nivo-chemo | Sint-chemo | Camre-chemo | Tori-chemo |
| --- | --- | --- | --- | --- | --- |
| Hypothyroidism | 0.111 | 0.063 | 0.147 | 0.118 | 0.058 |
| Pruritus | 0.044 | 0.097 | 0.028 | 0.070 | 0.117 |
| Hyperthyroidism | 0.052 | NA | 0.058 | 0.056 | 0.012 |
| Rash | 0.090 | 0.055 | 0.128 | 0.056 | 0.218 |
| Pneumonitis | 0.045 | 0.033 | 0.021 | 0.017 | 0.101 |
| Colitis | 0.027 | 0.004 | NA | NA | NA |
| Adrenal insufficiency | 0.010 | 0.009 | NA | 0.010 | NA |

# **Table S4. Sensitivity analyses of efficacy and safety.**

(1) OS; (2) PFS; (3) ORR; (4) TRAE (OS: overall survival; PFS: progression-free survival; ORR: objective response rate; TRAE: treatment-related adverse event)

(1) OS

| Characteristics | subgroups | No. of RCTs | No. of participants | MD(months) | 95% CI | Z | P(effect) | Chi^2^ | I^2^ | P(het) |
| --- | --- | --- | --- | --- | --- | --- | --- | --- | --- | --- |
| Publication year | 2020 | 1 | 507 | 1.35 | (-1.03, 3.73) | 1.11 | 0.27 | NA | NA | NA |
|  | 2021 | 2 | 2330 | 2.06 | (0.98, 3.14) | 3.73 | 0.0002 | 0.20 | 0% | 0.65 |
|  | 2022 | 4 | 2624 | 2.85 | (1.32, 4.37) | 3.66 | 0.0003 | 3.16 | 5% | 0.37 |
| Sample size | <600 | 2 | 1103 | 2.08 | (0.35, 3.81) | 2.35 | 0.02 | 0.77 | 0% | 0.38 |
|  | ≥600 | 5 | 4358 | 2.25 | (1.32, 3.18) | 4.75 | <0.00001 | 3.84 | 0% | 0.43 |
| Risk of bias | Low | 5 | 3235 | 2.37 | (1.17, 3.57) | 3.87 | 0.0001 | 4.18 | 4% | 0.38 |
|  | Some concerns | 2 | 2226 | 2.06 | (0.91, 3.21) | 3.52 | 0.0004 | 0.33 | 0% | 0.59 |
| Type of PD-1s | Pem-chemo | 2 | 1256 | 1.99 | (0.45, 3.53) | 2.54 | 0.01 | 0.48 | 0% | 0.49 |
|  | Nivo-chemo | 3 | 2950 | 1.94 | (0.85, 3.03) | 3.49 | 0.0005 | 0.78 | 0% | 0.68 |
|  | Sint-chemo | 1 | 659 | 5.50 | (1.63, 9.37) | 2.79 | 0.005 | NA | NA | NA |
|  | Camre-chemo | 1 | 596 | 2.90 | (0.37, 5.43) | 2.25 | 0.02 | NA | NA | NA |
| Ethnicity | Asian | 2 | 1320 | 2.17 | (0.14, 4.20) | 2.09 | 0.04 | 0.91 | 0% | 0.34 |
|  | Asian+White | 5 | 4141 | 2.22 | (1.33, 3.12) | 4.86 | <0.00001 | 3.72 | 0% | 0.44 |

(2) PFS

| Characteristics | subgroups | No. of RCTs | No. of participants | MD(months) | 95% CI | Z | P(effect) | Chi^2^ | I^2^ | P(het) |
| --- | --- | --- | --- | --- | --- | --- | --- | --- | --- | --- |
| Publication year | 2020 | 1 | 507 | 0.15 | (-0.88, 1.18) | 0.29 | 0.78 | NA | NA | NA |
|  | 2021 | 2 | 2330 | 1.01 | (0.54, 1.48) | 4.19 | <0.0001 | 0.04 | 0% | 0.84 |
|  | 2022 | 5 | 3138 | 1.16 | (0.70, 1.63) | 4.89 | <0.00001 | 4.13 | 3% | 0.39 |
| Sample size | <600 | 3 | 1617 | 0.77 | (0.29, 1.25) | 3.17 | 0.002 | 1.83 | 0% | 0.40 |
|  | ≥600 | 5 | 4358 | 1.16 | (0.75, 1.57) | 5.56 | <0.00001 | 3.92 | 0% | 0.42 |
| Risk of bias | Low | 6 | 3749 | 1.01 | (0.54, 1.47) | 4.26 | <0.0001 | 7.07 | 29% | 0.22 |
|  | Some concerns | 2 | 2226 | 1.03 | (0.42, 1.64) | 3.32 | 0.0009 | 0.14 | 0% | 0.71 |
| Type of PD-1s | Pem-chemo | 2 | 1256 | 0.70 | (-0.16, 1.56) | 1.59 | 0.11 | 2.17 | 54% | 0.14 |
|  | Nivo-chemo | 3 | 2950 | 1.11 | (0.51, 1.71) | 3.62 | 0.0003 | 1.98 | 0% | 0.37 |
|  | Sint-chemo | 1 | 659 | 2.15 | (0.70, 3.60) | 2.90 | 0.004 | NA | NA | NA |
|  | Camre-chemo | 1 | 596 | 1.00 | (0.19, 1.81) | 2.43 | 0.02 | NA | NA | NA |
|  | Tori-chemo | 1 | 514 | 0.90 | (0.17, 1.63) | 2.42 | 0.02 | NA | NA | NA |
| Ethnicity | Asian | 3 | 1834 | 1.01 | (0.47, 1.55) | 3.66 | 0.0003 | 2.02 | 1% | 0.36 |
|  | Asian+White | 5 | 4141 | 1.00 | (0.54, 1.46) | 4.30 | <0.0001 | 5.20 | 23% | 0.27 |

(3) ORR

| Characteristics | subgroups | No. of RCTs | No. of participants | MD(months) | 95% CI | Z | P(effect) | Chi^2^ | I^2^ | P(het) |
| --- | --- | --- | --- | --- | --- | --- | --- | --- | --- | --- |
| Publication year | 2020 | 1 | 507 | 1.60 | (1.12, 2.28) | 2.59 | 0.009 | NA | NA | NA |
|  | 2021 | 3 | 2980 | 1.62 | (1.33, 1.99) | 4.68 | <0.00001 | 3.46 | 42% | 0.18 |
|  | 2022 | 5 | 3138 | 1.93 | (1.57, 2.38) | 6.23 | <0.00001 | 8.05 | 50% | 0.09 |
| Sample size | <600 | 3 | 1617 | 1.73 | (1.41, 2.12) | 5.27 | <0.00001 | 1.39 | 0% | 0.50 |
|  | ≥600 | 6 | 5008 | 1.80 | (1.49, 2.17) | 6.11 | <0.00001 | 12.90 | 61% | 0.02 |
| Risk of bias | Low | 6 | 3749 | 1.81 | (1.56, 2.11) | 7.82 | <0.00001 | 6.29 | 20% | 0.28 |
|  | Some concerns | 3 | 2876 | 1.72 | (1.26, 2.35) | 3.41 | 0.0007 | 7.53 | 73% | 0.02 |
| Type of PD-1s | Pem-chemo | 2 | 1256 | 1.81 | (1.44, 2.28) | 5.07 | <0.00001 | 0.82 | 0% | 0.37 |
|  | Nivo-chemo | 3 | 2950 | 1.77 | (1.36, 2.31) | 4.23 | <0.0001 | 5.79 | 65% | 0.06 |
|  | Sint-chemo | 2 | 1309 | 1.75 | (0.99, 3.08) | 1.93 | 0.05 | 6.43 | 84% | 0.01 |
|  | Camre-chemo | 1 | 596 | 1.58 | (1.12, 2.23) | 2.61 | 0.009 | NA | NA | NA |
|  | Tori-chemo | 1 | 514 | 2.07 | (1.44, 2.97) | 3.95 | <0.0001 | NA | NA | NA |
| Ethnicity | Asian | 3 | 1834 | 1.66 | (1.36, 2.02) | 5.05 | <0.00001 | 2.12 | 6% | 0.35 |
|  | Asian+White | 5 | 4141 | 1.94 | (1.62, 2.31) | 7.37 | <0.00001 | 7.24 | 45% | 0.12 |

(4) TRAE

| Characteristics | subgroups | No. of RCTs | No. of participants | MD(months) | 95% CI | Z | P(effect) | Chi^2^ | I^2^ | P(het) |
| --- | --- | --- | --- | --- | --- | --- | --- | --- | --- | --- |
| Publication year | 2020 | 1 | 494 | 1.40 | (0.70, 2.80) | 0.95 | 0.34 | NA | NA | NA |
|  | 2021 | 2 | 2330 | 2.42 | (1.73, 3.39) | 5.17 | <0.00001 | 0.02 | 0% | 0.88 |
|  | 2022 | 5 | 3138 | 1.73 | (1.09, 2.76) | 2.31 | 0.02 | 4.84 | 17% | 0.30 |
| Sample size | <600 | 3 | 1604 | 1.79 | (0.79, 4.02) | 1.40 | 0.16 | 2.63 | 24% | 0.27 |
|  | ≥600 | 5 | 4358 | 2.07 | (1.56, 2.74) | 5.03 | <0.00001 | 4.32 | 7% | 0.36 |
| Risk of bias | Low | 5 | 3235 | 1.48 | (0.97, 2.26) | 1.82 | 0.07 | 4.30 | 0% | 0.51 |
|  | Some concerns | 2 | 2226 | 2.34 | (1.76, 3.12) | 5.80 | <0.00001 | 0.08 | 0% | 0.78 |
| Type of PD-1s | Pem-chemo | 2 | 1243 | 1.61 | (0.87, 2.97) | 1.51 | 0.13 | 0.72 | 0% | 0.40 |
|  | Nivo-chemo | 3 | 2950 | 2.13 | (1.55, 2.92) | 4.67 | <0.00001 | 2.44 | 18% | 0.30 |
|  | Sint-chemo | 1 | 659 | 0.98 | (0.31, 3.09) | 0.03 | 0.98 | NA | NA | NA |
|  | Camre-chemo | 1 | 596 | 5.14 | (1.12, 23.66) | 2.10 | 0.04 | NA | NA | NA |
|  | Tori-chemo | 1 | 514 | 1.00 | (0.14, 7.15) | 0.00 | 1.00 | NA | NA | NA |
| Ethnicity | Asian | 3 | 1834 | 1.71 | (0.66, 4.41) | 1.11 | 0.27 | 3.02 | 34% | 0.22 |
|  | Asian+White | 5 | 4128 | 2.10 | (1.63, 2.71) | 5.74 | <0.00001 | 3.77 | 0% | 0.44 |

# Table S5. GRADE approach.

| Table S5. GRADE approach. | | | | | | | | | | | | | |
| --- | --- | --- | --- | --- | --- | --- | --- | --- | --- | --- | --- | --- | --- |
|  | **Certainty assessment** | | | | | | **No of patients** | | **Effect** | |  |  | |
| No of studies | **Study design** | **Risk of bias** | **Inconsistency** | **Indirectness** | **Imprecision** | **Other considerations** | **ICIs combined with chemo** | **chemo** | **Relative (95% CI)** | **Absolute (95% CI)** | **Certainty** | **Importance** |  |
| Overall Survivals | | | | | | | | | | | | | |
| 8 | randomised trials | not serious | not serious | not serious | not serious | publication bias strongly suspected | 3054 | 3064 | - | MD **2.21 higher** (1.39 higher to 3.03 higher); HR 0.75 (0.69 to 0.81) | ⨁⨁⨁◯ Moderate | CRITICAL |  |
| Progress free survival | | | | | | | | | | | | | |
| 8 | randomised trials | not serious | not serious | not serious | not serious | publication bias strongly suspected | 3054 | 3064 | - | MD **1.00 higher** (0.68 higher to 1.32 higher); HR 0.66 (0.60 to 0.73) | ⨁⨁⨁◯ Moderate | IMPORTANT |  |
| Objective response rate | | | | | | | | | | | | | |
| 9 | randomised trials | not serious | serious | not serious | not serious | none | 1933/3311 (58.4%) | 1487/3314 (44.9%) | **OR 1.77** (1.55 to 2.03) | **142 more per 1,000** (from 109 more to 174 more) | ⨁⨁⨁◯ Moderate | IMPORTANT |  |
| Duration of response | | | | | | | | | | | | | |
| 7 | randomised trials | not serious | not serious | not serious | not serious | publication bias strongly suspected | 2172 | 2103 | - | MD **1.99 higher** (1.16 higher to 2.81 higher) | ⨁⨁⨁◯ Moderate | IMPORTANT |  |
| Treatment-related adverse events | | | | | | | | | | | | | |
| 8 | randomised trials | not serious | not serious | not serious | not serious | publication bias strongly suspected | 2863/2977 (96.2%) | 2764/2985 (92.6%) | **OR 1.99** (1.54 to 2.58) | **35 more per 1,000** (from 25 more to 44 more) | ⨁⨁⨁◯ Moderate | IMPORTANT |  |
| Serious adverse events | | | | | | | | | | | | | |
| 6 | randomised trials | not serious | not serious | not serious | not serious | none | 607/2343 (25.9%) | 404/2364 (17.1%) | **OR 1.71** (1.48 to 1.97) | **90 more per 1,000** (from 63 more to 118 more) | ⨁⨁⨁⨁ High | IMPORTANT |  |
| Immune-related adverse events | | | | | | | | | | | | | |
| 5 | randomised trials | not serious | very serious | not serious | not serious | publication bias strongly suspected | 657/1505 (43.7%) | 309/1507 (20.5%) | **OR 3.46** (1.81 to 6.60) | **267 more per 1,000** (from 113 more to 425 more) | ⨁◯◯◯ Very low | IMPORTANT |  |

# Supplementary S1. Search strategies.

(1) PubMed

#1 (Stomach Neoplasms [Mesh]) OR (Neoplasm, Stomach) OR (Stomach Neoplasm) OR (Neoplasms, Stomach) OR (Gastric Neoplasms) OR (Gastric Neoplasm) OR (Neoplasm, Gastric) OR (Neoplasms, Gastric) OR (Cancer of Stomach) OR (Stomach Cancers) OR (Gastric Cancer) OR (Cancer, Gastric) OR (Cancers, Gastric) OR (Gastric Cancers) OR (Stomach Cancer) OR (Cancer, Stomach) OR (Cancers, Stomach) OR (Cancer of the Stomach) OR (Gastric Cancer, Familial Diffuse)

#2 (Esophageal Neoplasms [Mesh]) OR (Esophageal Neoplasm) OR (Neoplasm, Esophageal) OR (Esophagus Neoplasm) OR (Esophagus Neoplasms) OR (Neoplasm, Esophagus) OR (Neoplasms, Esophagus) OR (Neoplasms, Esophageal) OR (Cancer of Esophagus) OR (Cancer of the Esophagus) OR (Esophagus Cancer) OR (Cancer, Esophagus) OR (Cancers, Esophagus) OR (Esophagus Cancers) OR (Esophageal Cancer) OR (Cancer, Esophageal) OR (Cancers, Esophageal) OR (Esophageal Cancers)

#3 (Esophagogastric Junction [Mesh]) OR (Junction, Esophagogastric) OR (Gastroesophageal Junction) OR (Gastroesophageal Junctions) OR (Junction, Gastroesophageal) OR (Junctions, Gastroesophageal)

#4 #1 OR #2 OR #3

#5 (Immune Checkpoint Inhibitors [Mesh]) OR (Checkpoint Inhibitors, Immune) OR (Immune Checkpoint Inhibitor) OR (Checkpoint Inhibitor, Immune) OR (Immune Checkpoint Blockers) OR (Checkpoint Blockers, Immune) OR (Immune Checkpoint Blockade) OR (Checkpoint Blockade, Immune) OR (Immune Checkpoint Inhibition) OR (Checkpoint Inhibition, Immune) OR (PD-L1 Inhibitors) OR (PD L1 Inhibitors) OR (PD-L1 Inhibitor) OR (PD L1 Inhibitor) OR (Programmed Death-Ligand 1 Inhibitors) OR (Programmed Death Ligand 1 Inhibitors) OR (PD-1-PD-L1 Blockade) OR (Blockade, PD-1-PD-L1) OR (PD 1 PD L1 Blockade) OR (CTLA-4 Inhibitors) OR (CTLA 4 Inhibitors) OR (CTLA-4 Inhibitor) OR (CTLA 4 Inhibitor) OR (Cytotoxic T-Lymphocyte-Associated Protein 4 Inhibitors) OR (Cytotoxic T Lymphocyte Associated Protein 4 Inhibitors) OR (Cytotoxic T-Lymphocyte-Associated Protein 4 Inhibitor) OR (Cytotoxic T Lymphocyte Associated Protein 4 Inhibitor) OR (PD-1 Inhibitors) OR (PD 1 Inhibitors) OR (PD-1 Inhibitor) OR (Inhibitor, PD-1) OR (PD 1 Inhibitor) OR (Programmed Cell Death Protein 1 Inhibitor) OR (Programmed Cell Death Protein 1 Inhibitors)

#6 nivolumab OR pembrolizumab OR camrelizumab OR sintilimab OR tislelizumab OR toripalimab OR penpulimab OR atezolizumab OR durvalumab OR avelumab OR tremelimumab OR ipilimumab

#7 #5 OR #6

#8 #4 AND #7

#9 (Randomized Controlled Trial [Publication Type]) OR (Trial [Publication Type])

#10 #8 and #9

(2) Web of Science

TS=(Stomach Neoplasms OR Neoplasm, Stomach OR Stomach Neoplasm OR Neoplasms, Stomach OR Gastric Neoplasms OR Gastric Neoplasm OR Neoplasm, Gastric OR Neoplasms, Gastric OR Cancer of Stomach OR Stomach Cancers OR Gastric Cancer OR Cancer, Gastric OR Cancers, Gastric OR Gastric Cancers OR Stomach Cancer OR Cancer, Stomach OR Cancers, Stomach OR Cancer of the Stomach OR Gastric Cancer, Familial Diffuse OR Esophageal Neoplasms OR Esophageal Neoplasm OR Neoplasm, Esophageal OR Esophagus Neoplasm OR Esophagus Neoplasms OR Neoplasm, Esophagus OR Neoplasms, Esophagus OR Neoplasms, Esophageal OR Cancer of Esophagus OR Cancer of the Esophagus OR Esophagus Cancer OR Cancer, Esophagus OR Cancers, Esophagus OR Esophagus Cancers OR Esophageal Cancer OR Cancer, Esophageal OR Cancers, Esophageal OR Esophageal Cancers OR Esophagogastric Junction OR Junction, Esophagogastric OR Gastroesophageal Junction OR Gastroesophageal Junctions OR Junction, Gastroesophageal OR Junctions, Gastroesophageal) AND TS=(Immune Checkpoint Inhibitors OR Checkpoint Inhibitors, Immune OR Immune Checkpoint Inhibitor OR Checkpoint Inhibitor, Immune OR Immune Checkpoint Blockers OR Checkpoint Blockers, Immune OR Immune Checkpoint Blockade OR Checkpoint Blockade, Immune OR Immune Checkpoint Inhibition OR Checkpoint Inhibition, Immune OR PD-L1 Inhibitors OR PD L1 Inhibitors OR PD-L1 Inhibitor OR PD L1 Inhibitor OR Programmed Death-Ligand 1 Inhibitors OR Programmed Death Ligand 1 Inhibitors OR PD-1-PD-L1 Blockade OR Blockade, PD-1-PD-L1 OR PD 1 PD L1 Blockade OR CTLA-4 Inhibitors OR CTLA 4 Inhibitors OR CTLA-4 Inhibitor OR CTLA 4 Inhibitor OR Cytotoxic T-Lymphocyte-Associated Protein 4 Inhibitors OR Cytotoxic T Lymphocyte Associated Protein 4 Inhibitors OR Cytotoxic T-Lymphocyte-Associated Protein 4 Inhibitor OR Cytotoxic T Lymphocyte Associated Protein 4 Inhibitor OR PD-1 Inhibitors OR PD 1 Inhibitors OR PD-1 Inhibitor OR Inhibitor, PD-1 OR PD 1 Inhibitor OR Programmed Cell Death Protein 1 Inhibitor OR Programmed Cell Death Protein 1 Inhibitors OR nivolumab OR pembrolizumab OR camrelizumab OR sintilimab OR tislelizumab OR toripalimab OR penpulimab OR atezolizumab OR durvalumab OR avelumab OR tremelimumab OR ipilimumab) AND TS=(Randomized Controlled Trial OR Trial)

(3) Cochrane Library

#1 (Stomach Neoplasms OR Neoplasm, Stomach OR Stomach Neoplasm OR Neoplasms, Stomach OR Gastric Neoplasms OR Gastric Neoplasm OR Neoplasm, Gastric OR Neoplasms, Gastric OR Cancer of Stomach OR Stomach Cancers OR Gastric Cancer OR Cancer, Gastric OR Cancers, Gastric OR Gastric Cancers OR Stomach Cancer OR Cancer, Stomach OR Cancers, Stomach OR Cancer of the Stomach OR Gastric Cancer, Familial Diffuse OR Esophageal Neoplasms OR Esophageal Neoplasm OR Neoplasm, Esophageal OR Esophagus Neoplasm OR Esophagus Neoplasms OR Neoplasm, Esophagus OR Neoplasms, Esophagus OR Neoplasms, Esophageal OR Cancer of Esophagus OR Cancer of the Esophagus OR Esophagus Cancer OR Cancer, Esophagus OR Cancers, Esophagus OR Esophagus Cancers OR Esophageal Cancer OR Cancer, Esophageal OR Cancers, Esophageal OR Esophageal Cancers OR Junction, Esophagogastric OR Gastroesophageal Junction OR Gastroesophageal Junctions OR Junction, Gastroesophageal OR Junctions, Gastroesophageal):ab,ti,kw

#2 (Immune Checkpoint Inhibitors OR Checkpoint Inhibitors, Immune OR Immune Checkpoint Inhibitor OR Checkpoint Inhibitor, Immune OR Immune Checkpoint Blockers OR Checkpoint Blockers, Immune OR Immune Checkpoint Blockade OR Checkpoint Blockade, Immune OR Immune Checkpoint Inhibition OR Checkpoint Inhibition, Immune OR PD-L1 Inhibitors OR PD L1 Inhibitors OR PD-L1 Inhibitor OR PD L1 Inhibitor OR Programmed Death-Ligand 1 Inhibitors OR Programmed Death Ligand 1 Inhibitors OR PD-1-PD-L1 Blockade OR Blockade, PD-1-PD-L1 OR PD 1 PD L1 Blockade OR CTLA-4 Inhibitors OR CTLA 4 Inhibitors OR CTLA-4 Inhibitor OR CTLA 4 Inhibitor OR Cytotoxic T-Lymphocyte-Associated Protein 4 Inhibitors OR Cytotoxic T Lymphocyte Associated Protein 4 Inhibitors OR Cytotoxic T-Lymphocyte-Associated Protein 4 Inhibitor OR Cytotoxic T Lymphocyte Associated Protein 4 Inhibitor OR PD-1 Inhibitors OR PD 1 Inhibitors OR PD-1 Inhibitor OR Inhibitor, PD-1 OR PD 1 Inhibitor OR Programmed Cell Death Protein 1 Inhibitor OR Programmed Cell Death Protein 1 Inhibitors OR nivolumab OR pembrolizumab OR camrelizumab OR sintilimab OR tislelizumab OR toripalimab OR penpulimab OR atezolizumab OR durvalumab OR avelumab OR tremelimumab OR ipilimumab):ab,ti,kw

#3 #1 AND #2

(4) Embase

#1 ‘Stomach Neoplasms’:ab,ti OR ‘Stomach Neoplasm’:ab,ti OR ‘Neoplasms, Stomach’:ab,ti OR ‘Gastric Neoplasms’:ab,ti OR ‘Gastric Neoplasm’:ab,ti OR ‘Neoplasm, Gastric’:ab,ti OR ‘Neoplasms, Gastric’:ab,ti OR ‘Cancer of Stomach’:ab,ti OR ‘Stomach Cancers’:ab,ti OR ‘Gastric Cancer’:ab,ti OR ‘Cancer, Gastric’:ab,ti OR ‘Cancers, Gastric’:ab,ti OR ‘Gastric Cancers’:ab,ti OR ‘Stomach Cancer’:ab,ti OR ‘Cancer, Stomach’:ab,ti OR ‘Cancers, Stomach’:ab,ti OR ‘Cancer of the Stomach’:ab,ti OR ‘Gastric Cancer, Familial Diffuse’:ab,ti

#2 ‘Esophageal Neoplasm’:ab,ti OR ‘Neoplasm, Esophageal’:ab,ti OR ‘Esophagus Neoplasm’:ab,ti OR ‘Esophagus Neoplasms’:ab,ti OR ‘Neoplasm, Esophagus’:ab,ti OR ‘Neoplasms, Esophagus’:ab,ti OR ‘Neoplasms, Esophageal’:ab,ti OR ‘Cancer of Esophagus’:ab,ti OR ‘Cancer of the Esophagus’:ab,ti OR ‘Esophagus Cancer’:ab,ti OR ‘Cancer, Esophagus’:ab,ti OR ‘Cancers, Esophagus’:ab,ti OR ‘Esophagus Cancers’:ab,ti OR ‘Esophageal Cancer’:ab,ti OR ‘Cancer, Esophageal’:ab,ti OR ‘Cancers, Esophageal’:ab,ti OR ‘Esophageal Cancers’:ab,ti

#3 ‘Junction, Esophagogastric’:ab,ti OR ‘Gastroesophageal Junction’:ab,ti OR ‘Gastroesophageal Junctions’:ab,ti OR ‘Junction, Gastroesophageal’:ab,ti OR ‘Junctions, Gastroesophageal’ :ab,ti

#4 #1 OR #2 OR #3

#5 ‘Immune Checkpoint Inhibitors’:ab,ti OR ‘Checkpoint Inhibitors, Immune’:ab,ti OR ‘Immune Checkpoint Inhibitor’:ab,ti OR ‘Checkpoint Inhibitor, Immune’:ab,ti OR ‘Immune Checkpoint Blockers’:ab,ti OR ‘Checkpoint Blockers, Immune’:ab,ti OR ‘Immune Checkpoint Blockade’:ab,ti OR ‘Checkpoint Blockade, Immune’:ab,ti OR ‘Immune Checkpoint Inhibition’:ab,ti OR ‘Checkpoint Inhibition, Immune’:ab,ti OR ‘PD-L1 Inhibitors’:ab,ti OR ‘PD L1 Inhibitors’:ab,ti OR ‘PD-L1 Inhibitor’:ab,ti OR ‘PD L1 Inhibitor’:ab,ti OR ‘Programmed Death-Ligand 1 Inhibitors’:ab,ti OR ‘Programmed Death Ligand 1 Inhibitors’:ab,ti OR ‘PD-1-PD-L1 Blockade’:ab,ti OR ‘Blockade, PD-1-PD-L1:ab,ti OR PD 1 PD L1 Blockade’:ab,ti OR ‘CTLA-4 Inhibitors’:ab,ti OR ‘CTLA 4 Inhibitors’:ab,ti OR ‘CTLA-4 Inhibitor’:ab,ti OR ‘CTLA 4 Inhibitor’:ab,ti OR ‘Cytotoxic T-Lymphocyte-Associated Protein 4 Inhibitors’:ab,ti OR ‘Cytotoxic T Lymphocyte Associated Protein 4 Inhibitors’:ab,ti OR ‘Cytotoxic T-Lymphocyte-Associated Protein 4 Inhibitor’:ab,ti OR ‘Cytotoxic T Lymphocyte Associated Protein 4 Inhibitor’:ab,ti OR ‘PD-1 Inhibitors’:ab,ti OR ‘PD 1 Inhibitors’:ab,ti OR ‘PD-1 Inhibitor’:ab,ti OR ‘Inhibitor, PD-1’:ab,ti OR ‘PD 1 Inhibitor:ab,ti OR Programmed Cell Death Protein 1 Inhibitor’:ab,ti OR ‘Programmed Cell Death Protein 1 Inhibitors’:ab,ti OR ‘nivolumab’:ab,ti OR ‘pembrolizumab’:ab,ti OR ‘camrelizumab’:ab,ti OR ‘sintilimab’:ab,ti OR ‘tislelizumab’:ab,ti OR ‘toripalimab’:ab,ti OR ‘penpulimab’:ab,ti OR ‘atezolizumab’:ab,ti OR ‘durvalumab’:ab,ti OR ‘avelumab’:ab,ti OR ‘tremelimumab’:ab,ti OR ‘ipilimumab’ :ab,ti

#4 AND #5
